# Seasonal Variation of Mycosporine-Like Amino Acids in Three Subantarctic Red Seaweeds

**DOI:** 10.3390/md18020075

**Published:** 2020-01-24

**Authors:** Jocelyn Jofre, Paula S. M. Celis-Plá, Félix L. Figueroa, Nelso P. Navarro

**Affiliations:** 1 Laboratorio de Ecofisiología y Biotecnología de Algas (LEBA), Facultad de Ciencias, Universidad de Magallanes, Punta Arenas 620000, Chile; jocelyn.jofre@umag.cl; 2 Laboratory of Aquatic Environmental Research, Center of Advanced Studies, Universidad de Playa Ancha, Traslaviña 450, Viña del Mar 581782, Chile; paulacelispla@upla.cl; 3 HUB-AMBIENTAL UPLA, Vicerrectoría de Investigación Postgrado e Innovación, Universidad de Playa Ancha, Av. Carvallo 270, Valparaíso 2340000, Chile; 4 Universidad de Málaga, Instituto Universitario de Biotecnología y Desarrollo Azul (IBYDA), Departamento de Ecología, Facultad de Ciencias, 29071 Malaga, Spain; felix_lopez@uma.es; 5 Centro FONDAP de Investigación en Dinámica de Ecosistemas Marinos de Altas Latitudes (IDEAL), Punta Arenas 620000, Chile; 6 Network for Extreme Environments Research, NEXER-Universidad de Magallanes, casilla 113-D, Punta Arenas 620000, Chile

**Keywords:** *Corallina*, *Iridaea*, Mycosporine-like amino acids, *Nothogenia*, red algae

## Abstract

UV-absorbing compounds, such as mycosporine-like amino acids (MAAs), are a group of secondary metabolites present in many marine species, including red seaweeds. In these organisms, the content and proportion of the composition of MAAs vary, depending on the species and several environmental factors. Its high cosmetic interest calls for research on the content and composition of MAAs, as well as the dynamics of MAAs accumulation in seaweeds from different latitudes. Therefore, this study aimed to survey the content of UV-absorbing MAAs in three Subantarctic red seaweeds during a seasonal cycle. Using spectrophotometric and HPLC techniques, the content and composition of MAAs of intertidal *Iridaea tuberculosa*, *Nothogenia fastigiate*, and *Corallina officinalis* were assessed. Some samples were also analyzed using high-resolution mass spectrometry coupled with HPLC-ESI-MS in order to identify more precisely the MAA composition. *I. tuberculosa* exhibited the highest MAA values (above 1 mg g^−1^ of dried mass weight), while *C. officinalis* showed values not exceeding 0.4 mg g^−1^. Porphyra-334 was the main component in *N.* *fastigiata*, whereas *I. tuberculosa* and *C.* *officinalis* exhibited a high content of palythine. Both content and composition of MAAs varied seasonally, with high concentration recorded in different seasons, depending on the species, i.e., winter (*I. tuberculosa*), spring (*N. fastigiata*), and summer (*C. officinalis*). HPLC-ESI-MS allowed us to identify seven different MAAs. Two were recorded for the first time in seaweeds from Subantarctic areas (mycosporine-glutamic acid and palythine-serine), and we also recorded an eighth UV-absorbing compound which remains unidentified.

## 1. Introduction

Recovery of the ozone layer is expected by the end of this century. Nevertheless, several factors could still contribute to the increase of ultraviolet radiation (UVR), including the decrease of aerosols and cloud cover interacting with climate change [1,2]. Thus, in the expected scenario, photoprotection of the biosphere will still be relevant [1,3]. Marine primary producers possess several compounds that act as photoprotectors against excess solar radiation. Among these compounds, mycosporine-like amino acids (MAAs) are widespread among different taxonomic groups from both freshwater and marine environments [4,5,6,7,8]. In seaweeds, MAAs are related to different functions but especially photoprotection by the ability of these compounds to absorb UVR harmful to human health (UV-B, λ = 280–315 nm and UV-A, λ = 315–400 nm). These UV-screening compounds have also been described as a reservoir of nitrogen (N), osmotic regulation, and antioxidant activity [9,10,11]. MAA compounds can be used as N reservoir whenever sources of N are reduced via an anticipatory strategy, as has been suggested for other accessory pigments, e.g., the production of phycobiliproteins under stress [12]. In addition, these compounds are considered as indicators of biological stress [13,14].

The accumulation of MAAs in seaweeds varies, depending on the species, location, and environment in which they are growing [15,16,17], with concentrations up to 14 mg g^−1^ dry weight [8,18]. MAAs content in seaweeds has been reported at different water depths [16] and geographical areas [15,16,19], and, in general, the highest MAA content often correlates with the degree of exposure to solar radiation [15]. In this context, species occurring in the upper intertidal zone and at low latitude while exposed to high UV solar radiation have high MAA concentration when compared to species from the subtidal zone and high latitude [20,21]. In terms of MAA accumulation and induction patterns after exposure to different radiation conditions, the red seaweeds have been classified into three different physiological groups, according to [16]: (i) species with no capacity for MAA biosynthesis; (ii) species with a constant and constitutive MAA composition at relatively high concentration, irrespective of environmental conditions; and (iii) species with a basic MAA concentration adjusted according to environmental conditions [16]. In the latter group, synthesis of MAAs could be influenced by changes in quality and quantity of environmental radiation (UV-A, UV-B, and/or Photosynthetically Active Radiation [PAR], especially blue light) [22,23,24,25] and by other environmental variables, such as salinity, temperature, and desiccation [26,27]. Although about 35 different MAAs have been identified, their diversity in red seaweeds is mainly reduced to only seven compounds, including mycosporine-glycine, porphyra-334, asterina-330, shinorine, palythinol, palythine, and palythene. Thus, they can cover a broad spectrum of UV radiation absorption from 310 nm (mycosporine-glycine) to 360 nm (palythene) according to [8,19]. Additionally, usujirene (λ_max_ at 357 nm) and catenelline (λ_max_ at 334 nm) have been reported in a few species, such as *Palmaria palmata*, *Porphyra yezoensis*, *Gracilaria tenuifrons*, and *Catenella repens* [28,29]. Advances in technology have revealed new MAAs, not only in seaweeds but also in different marine and terrestrial organisms [18,19,30,31].

Owing to the physicochemical features of low molecular weight, absorption in the UV region [4,32], high melting point, solubility in water and organic solvents [33], and extreme stability in a wide range of pH and temperature conditions [11], these compounds have sparked considerable interest among different biotechnological industries. MAAs can effectively and rapidly transform absorbed UV into harmless thermal energy with no subsequent loss of protective power [34]. This affords MAAs with potential application in the prevention and therapeutic treatment of diseases and disorders related to free-radical production and UV exposure in humans [35,36]. However, over the past few years, other properties such as immuno-stimulation, cell proliferation, elastin and hyaluronic acid, as well as protection of components of dermis and collagen, have made these compounds like *Polipodium leucotomos* extract, an oral photoprotector, useful in the cosmeceutical industry [37,38]. MAAs are also nontoxic and biodegradable products, making them safe for use in health and beauty products. Currently, the use of MAAs from seaweeds in new biotechnological products has not been completely explored, most likely a result of the low concentration of such compounds in these organisms [8]. In this context, the search for species with high MAAs content or the ability to synthesize these compounds is an unfinished task. Furthermore, using methodologically advanced tools to identify MAAs could reveal compounds not reported before in seaweed species. Consequently, species not previously important owing to their low MAAs content could become newly viable by the presence of some previously unknown MAA found in nature. Therefore, the present study aimed to survey the seasonal variation of MAAs content and composition in three intertidal red seaweed species (*Nothogenia fastigiata*, *Iridaea tuberculosa*, and *Corallina officinalis*) from the Magellan Strait of Chilean Patagonia. Although these species share the intertidal habitat, they differ in relation to their exposure to solar radiation and in their abundance during the year. In the case of the calcareous alga *Corallina officinalis*, light scattering could influence the accumulation pattern of MAAs in ways different from that of non-calcareous algae. Thus, we hypothesized that these species exhibit a different seasonal dynamic in total MAA concentration and even in MAA composition.

## 2. Results

### 2.1. Solar Irradiance and Temperature

**Figure 1:**
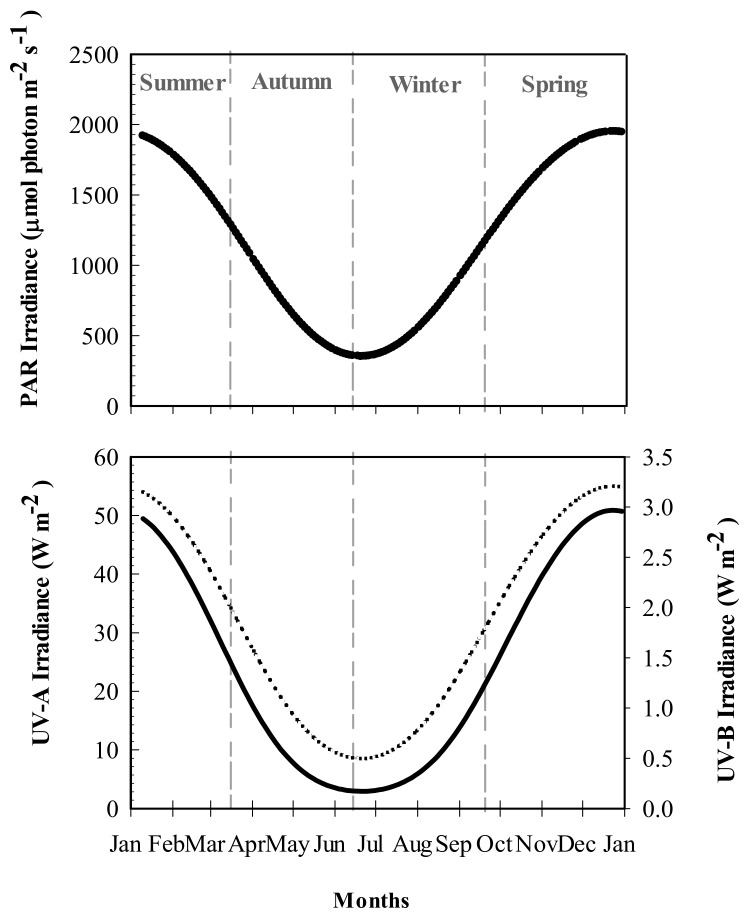
**Photosynthetically Active Radiation** (PAR: λ = 400–700 nm ) and UVR irradiances recorded at Bahía Mansa, Magellan Strait. UV-A (λ = 320–400 nm: dotted line), UV-B (λ = 290–320 nm: black solid line), and PAR values were obtained from the tropospheric ultraviolet visible model (TUV), considering measurements made around 17:00 h every 2 days during 2018.

The mean surface water temperature recorded in Bahía Mansa varied between 6.7 °C in winter-spring to 8.7 °C in summer. Seasonal differences in PAR and UV levels were recorded, with maximum values in summer (2.9 W m^−2^, 55 W m^−2^ and 1950 μmol photon m^−2^ s^−1^ of UV-A, UV-B, and PAR, respectively) and the lowest values (0.17 W m^−2^, 8 W m^−2^, and 350 μmol photon m^−2^ s^−1^ of UV-B, UV-A and PAR, respectively) observed in winter (Figure 1).

### 2.2. Seasonal Variation in Total MAA Content and MAA Composition

**Figure 2:**
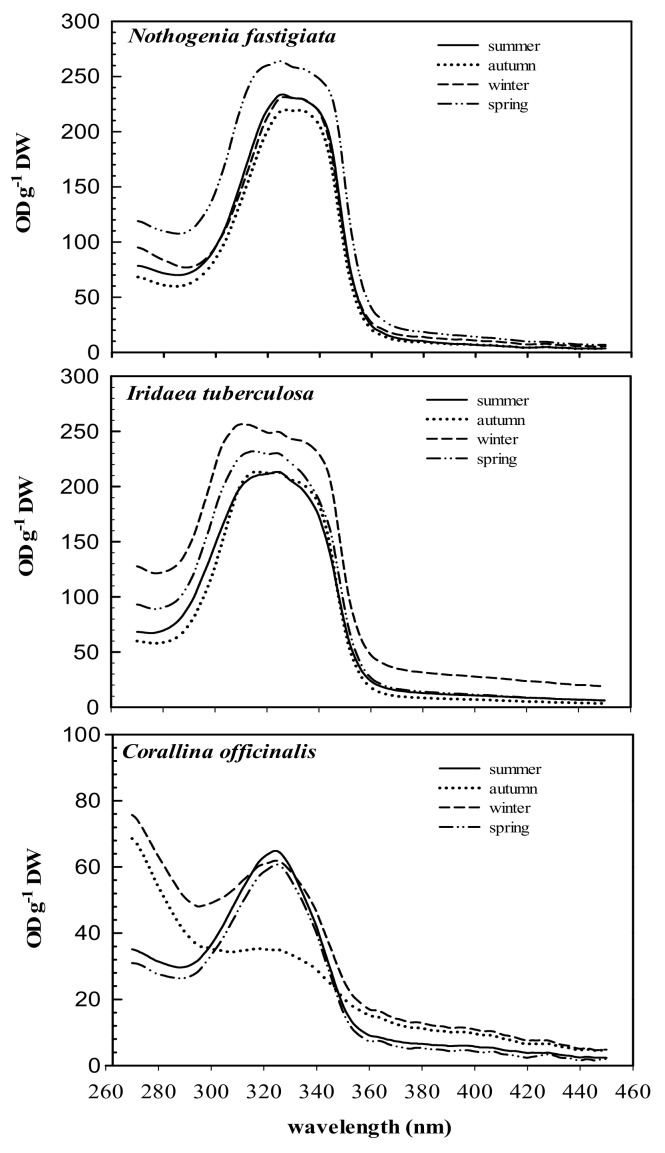
Absorption characteristics (OD g^−1^ DW) of 20% methanol extracts of *Nothogenia fastigiata, Iridaea tuberculosa*, and *Corallina officinalis* collected from Bahía Mansa, Magellan Strait, Chilean Patagonia, during summer, autumn, winter, and spring. Each curve is an average of five measurements.

The absorption spectra of 20% methanol extract from the three species and their variation throughout the year are shown in Figure 2. The three species analyzed exhibited absorption ranging between 290 and 360 nm, while maximal absorption peaks ranged from 311 to 327 nm. In high-performance liquid chromatography (HPLC) analyses, chromatograms showed the presence of MAAs, which varied according to species. Total MAA content in *Nothogenia fastigiata* ranged from 0.6 to 1.6 mg g^−1^ of dried mass weight. *Iridaea tuberculosa* exhibited the highest values of MAAs (above 1 mg g^−1^), while *Corallina officinalis* showed values not exceeding 0.4 mg g^−1^. 

**Figure 3:**
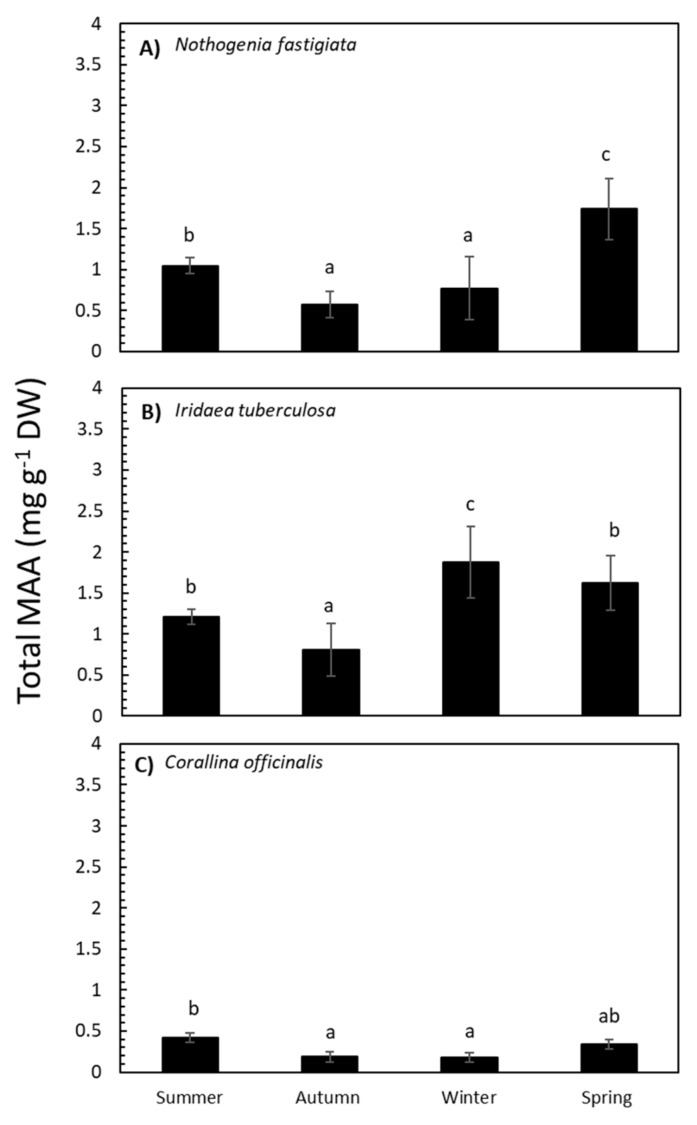
Total mycosporine-like amino acid (MAA) content expressed as mg g^−1^ DW (mean values ± SE, n = 3) in *Nothogenia fastigiata* (**A**), *Iridaea tuberculosa* (**B**), and *Corallina officinalis* (**C**) collected from Bahía Mansa, Magellan Strait, Chilean Patagonia, during summer, autumn, winter, and spring. Lowercase letters on the graph represent results of the Student Newman–Keuls Test (*p* <0.05); different letters refer to significant differences between mean values.

The total MAA content in *N. fastigiata* was significantly different (*p* <0.05) relative to seasonality (Table S1 and Figure 3). The MAA content was higher in spring and decreased in summer, autumn, and winter seasons. In *I. tuberculosa*, MAA content significantly increased in algae collected in winter compared to algae collected in spring and summer, with lower values in autumn (Table S1 and Figure 3). In addition, in *C. officinalis*, the highest accumulation of MAAs was observed in summer (Table S1 and Figure 3). Total MAA content was positively correlated with solar radiation in the case of *N. fastigiata* and *C. officinalis* but not in *I. tuberculosa* (Table S2A–C).

**Figure 4:**
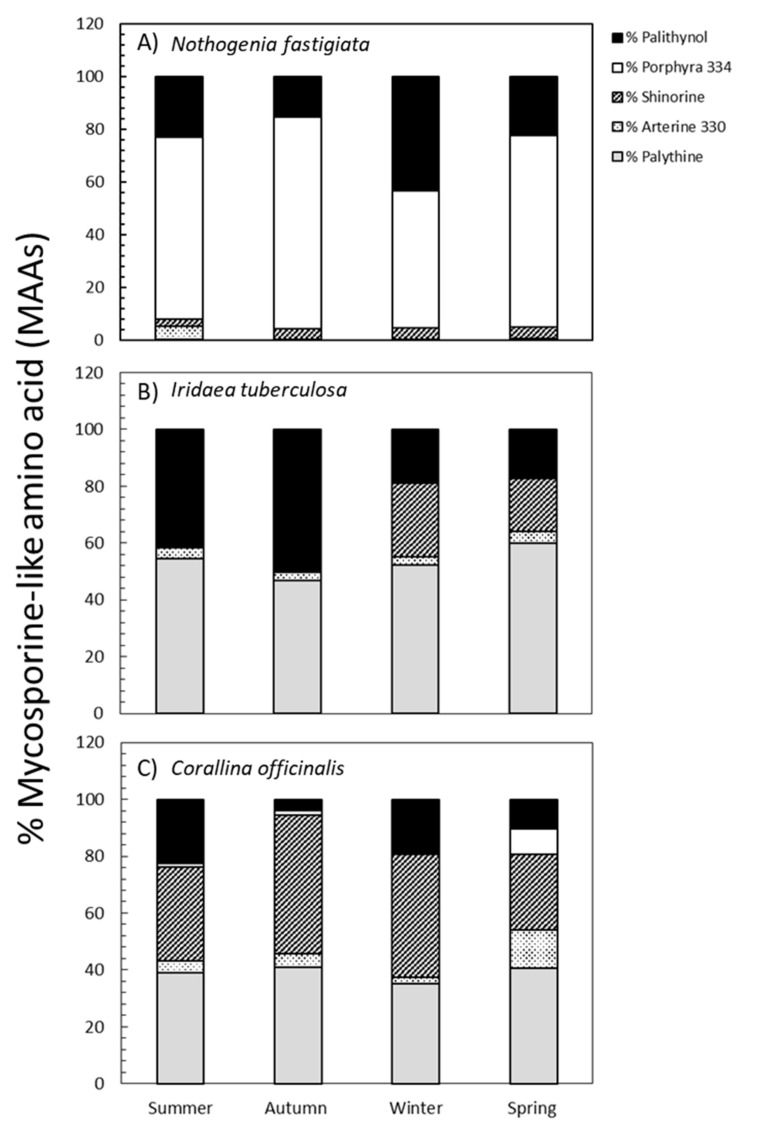
Percentages of palythine (grey bars), asterina-330 (plot points), shinorine (plot lines bars), porphyra-334 (white bars), and palythinol (black bars) (mean values, n = 3) in *Nothogenia fastigiata* (**A**), *Iridaea tuberculosa* (**B**), and *Corallina officinalis* (**C**) collected from Bahía Mansa, Magellan Strait, Chilean Patagonia, during summer, autumn, winter, and spring.

By comparing the absorption spectra and retention times with various well-characterized secondary standards, we were able to identify five different MAAs in three species, as follows: palythinol, porphyra-334, shinorine, asterina-330, and palythine. The percentages of each MAA relative to total MAA content are shown in Figure 4. The percentages of each MAA varied according to species and season of collection are shown in Table S3. In *N. fastigiata*, the percentage of palythinol varied between 15% in autumn and 43% in winter, whereas porphyra-334 exhibited values ranging from 50% in winter to 80% in autumn, and shinorine showed lower values (from 2% to 4% in summer and spring, respectively). Furthermore, the presence of asterina-330 (4%) and palythine (0.3%) was observed only in samples collected during summer (Figure 4). In *I. tuberculosa*, the percentage of palythinol increased significantly from 18% in winter-spring to 55% in summer-autumn conditions, while shinorine was only present in the winter-spring period (ranging from 25% to 18%, respectively). In addition, the percentage of palythine was higher in spring (60%) and summer (55%) (Figure 4). Palythine (40%), Palythinol (35%), and Shinorine (15%) were also the most representative MAAs in *C. officinalis*, whereas asterina-330 and porphyra-334 made up only a small proportion (4% and 2%, respectively). These last two MAAs exhibited an increase in spring (Figure 4).

### 2.3. Identification of MAAs under HPLC-ESI-MS

In order to corroborate the identification of MAAs and because the same samples exhibited peaks not possible to identify using secondary MAA standards, they were analyzed under HPLC-ESI-MS. This analysis allowed us to identify seven different MAAs: shinorine (λ_max_: 334 nm; *m*/*z*: 333.1292), porphyra-334 (λ_max_: 334 nm; *m*/*z*: 347.1449), asterina-330 (λ_max_: 330nm; *m*/*z*: 289.1394), palythinol (λ_max_: 332 nm; *m*/*z*: 303.1551), palythine-serine (λ_max_: 320nm; *m*/*z*: 275.1238), mycosporine-glycine (λ_max_: 310nm; *m*/*z*: 246.0972), mycosporine-glutamic acid (λ_max_: 311nm; *m*/*z*: 318.1183), and an eighth UV-absorbing compound which remains unidentified (λ_max_: 330 nm; *m*/*z*: 261.1445) (Table 1 and Figures S1–S15). All of these MAAs were recorded in *I. tuberculosa*, while only four of them were identified in *N. fastigiata* and *C. officinalis*.

## 3. Discussion

The present research provides information on the presence of MAAs in red seaweeds from the Subantarctic region with qualitative and quantitative data on three conspicuous species. This study reveals that the contents and composition of UV-absorbing MAAs experience strong seasonal changes in the three intertidal species. These changes are probably controlled by various abiotic factors, principally by the solar radiation regime and nitrate regime as shown in both laboratory [39,40,41,42] and field [43,44].

### 3.1. The Environmental Context and Seasonal Variation of MAAs 

The site of sample collection (Bahía Mansa, Magellan Strait in the Subantarctic region) exhibited wide seasonal difference in PAR and UV levels, with maximum values in summer and lowest values in winter. Additionally, this latitude in the Southern Hemisphere is exposed to an increase of biologically harmful UVR because of ozone depletion [2], which takes place in early spring. On the other hand, the mean surface water temperature recorded in Bahía Mansa varied between 6.7 °C in winter-spring to 8.7 °C in summer. However, at local level, in the intertidal of Magellan Strait, other factors can vary according to tide and season; this environmental variation could impose constraints on the physiology and growth of seaweeds. In the intertidal zone of Magellan Strait, the species *Nothogenia fastigiata* and *Iridaea tuberculosa* grow well in the mid-intertidal, even when exposed to high solar radiation and desiccation. However, *I. tuberculosa* occupies more shaded and wet places (e.g., cracks), and *Corallina officinalis* occurs in the lower intertidal where variations in environmental conditions could be less severe.

MAA compounds were presented throughout the year in the three species; however, the concentration and composition of MAAs followed strong seasonal changes. In the case of *N. fastigiata* and *C. officinalis*, MAA content increased during spring and summer, respectively. This seasonal pattern in MAA content with maximum concentrations under high incident levels of solar radiation is consistent with the idea that these compounds are synthesized and accumulated in response to solar irradiance stress (mainly UV). In fact, MAA content in these two species correlated positively with solar radiation. Additionally, the fact that ozone depletion takes place particularly in the Southern Hemisphere, which results in an increase of biologically harmful UVR reaching the Earth’s surface, magnifies the importance of MAAs in algae from these regions. We know that mycosporine-glycine (UV-B-absorbing MAA) increased to the detriment of the UV-A-absorbing MAAs in the red alga *Mazzaella laminarioides* during a period of ozone depletion in southern Chile, indicating that this seaweed can acclimate to increased UV-B radiation [45].

This is one of a few studies reporting concentrations and composition of MAAs relative to seasonal changes in seaweed species from high latitudes. Seasonal variation with high MAA content correlating with high solar irradiance was reported in the red alga *Gracilaria vermiculophylla* [46] cultured outdoors and planktonic organisms from lakes [47,48]. Furthermore, it was reported that UV-absorbing MAAs and antioxidant enzyme activities experience strong seasonal changes in *Palmaria palmata* and *Devaleraea ramentacea* from the Kongsfjorden on Spitsbergen. Both species exhibited a significant increase in the concentration of MAAs coinciding with the increase in underwater radiation during sea ice breakup in summer [49]. However, although a seasonal variation in the MAA concentration was reported in the seaweed *Pyropia plicata* from New Zealand, the increased total MAA content was not correlated with solar radiation [18]. In fact, the authors observed that fronds collected from April to August (autumn-winter period) exhibited the highest MAA content, while fronds collected from August to November (spring) showed a strong decrease in MAA content. This last report is consistent with our study with respect to *I. tuberculosa* data, which shows high total MAA concentration in winter.

According to the classification of Hoyer et al. [16,24], *N. fastigiata*, *I. tuberculosa*, and *C. officinalis* can be characterized as seaweeds with a basic MAA concentration that is adjusted according to environmental conditions. However, each species exhibited high MAA content in different periods of the year: *I. tuberculosa* in winter, *N. fastigiata* in spring, and *C. officinalis* in summer. This difference could be attributed to local environmental factors associated with the microhabitat where each species grows in the intertidal, which could, in turn, reflect MAA composition and accumulation. In a given seasonal period, it has been suggested that some UV-absorbing secondary metabolites (e.g., phenolic compounds) have a stronger correlation with local environmental factors than large-scale factors (e.g., months, seasons, latitude) [50,51]. The content of MAAs in red seaweeds from the Chilean coast (mid latitude) was higher in organisms collected from the eulittoral area compared to the sublittoral area in relation to higher daily integrated irradiance and other stress conditions, such as desiccation events [17]. Other variables, such as nitrogen supply in upwelling coastal areas of Brazil, produce an increase of MAAs, whereas in areas with high irradiance and nitrate-poor waters, the level of MAAs decreased [44]. In the same context, in the upper intertidal, *N. fastigiata* is exposed to higher solar irradiance and thermal stress during spring and summer compared to the other two studied species, and for this reason, an increase of MAA content would take place during these seasons. Additionally, porphyra-334 is the quantitatively most important MAA in *N. fastigiata* at over 60% in all seasons, except in winter, with palythinol between 15% and 40%. The presence of porphyra-334 as the main component is reported in species that preferentially are often exposed to high solar radiation and grow in the upper littoral to supralittoral zones [16,18,19]. On the other hand, *N. fastigiata* collected during summer in Valdivia (Chile) exhibited higher MAA content (3.8 mg g^-1^ of dried mass weight) and different composition as follows: porphyra-334 (80%), shinorine (10%), and palythinol, asterina-330, palythine and mycosporine-glycine with less than 4% each [17]. This suggests that concentration and composition could vary with latitude and that this variation could be related to levels of solar irradiance. Interestingly, in our study, even though total MAA concentration of *N. fastigiata* decreased from spring to summer, asterina-330 was still synthetized (5%) in summer. This type of MAA has an important antioxidant activity against lipid peroxidation, similar to porphyra-334 [11], which could be important to quenching oxygen reactive species promoted by light excess.

*Iridaea tuberculosa* showed the highest MAA content among the studied species. This species accumulated more MAAs during winter. On the other hand, unlike *N. fastigiata*, *I. tuberculosa* has palythine (more than 50%) as the most representative MA but also palythinol (ranging from 18% to 50%). This is consistent with the report that palythine (and shinorine) are usually the dominant MAAs in red seaweed species inhabiting more shaded places (e.g., subtidal) [16]. The high MAA content in *Pyropia plicata* during winter was related to nitrogen availability in seawater [18] on account of the positive correlation between N content and MAAs observed in several species [9,25,40,45,52]. However, seawater of the Magellan Strait can be considered mesotrophic waters since NO_3_^-^ concentration is rather uniform at about 0.005 mM, and no annual significant variation has ever been reported [53]. Thus, the induction and synthesis of MAAs in winter in *I. tuberculosa* could be influenced not only by specific wavelengths during short light periods but also by any possible endogenous biological controlling factor. It should be noted that *I. tuberculosa* is more abundant during autumn and winter seasons.

*N. fastigiata* and *I. tuberculosa* decreased their concentration of MAAs in summer when compared to previous seasons. Both species exhibited a high concentration of palythinol, which varied differently in both species during the year. Specifically, the former exhibited a high palythinol concentration in winter, while the latter showed high concentration in the summer-autumn seasons. In addition, short-term variation of MAAs was reported. For example, *Porphyra columbina* increased its MAA content 3 h before high solar radiation [52]. In studies on action spectra for the induction of MAAs in *Chondrus crispus* from Helgoland (Germany), short wavelength UV-B exhibited the highest quantum efficiency in its synthesis as well as in asterina-330 and palythine [54].

The calcareous *C. officinalis* exhibited the lowest total MAA concentration among the studied species, ranging from 0.1 to 0.4 mg g^-1^ of dried mass weight. The low total MAA content could be a reflection of its acclimation to low radiation levels. On the other hand, it should be noted that the cell wall of *C. officinalis* contains calcium carbonate, which effectively absorbs UV radiation, thus minimizing the exposure of important biomolecules in that alga [13,55]. MAA concentration varied seasonally, with high levels being observed during spring-summer and the lowest recorded during autumn-winter. The most abundant MAAs detected in this species were shinorine (25%–50%) and palythine (approx. 35%–40%); other MAAs, such as asterina-330, were also present (4%–6%). Shinorine was also the main and only MAA reported in *Corallina officinalis* from Argentinian Patagonia [55]. Similar results were reported in *Ellisolandia elongata* (before *Corallina elongata*), with shinorine (50% to 60%) and palythine (approx. 40%) being the most abundant MAAs, while other MAAs, such as asterina-330, were present in trace amounts [14]. On the other hand, the inventory and percentage of MAAs in *C. officinalis* are consistent with those of *C. officinalis* from New Zealand [19]. Interestingly, asterina-330 increased in spring (6%) when compared to the previous season (3.8%). Similarly, porphyra-334 was synthetized from zero (in winter) to 8% in spring to the detriment of shinorine. Under light stress, all of these MAAs could prevent photodamage by their UV screening and antioxidant capacity [11].

### 3.2. MAA Composition by HPLC-ESI-MS

Seven different MAAs were identified using HPLC-ESI-MS, with palythine-serine and mycosporine-glutamic being recorded for the first time in seaweed of Subantarctic ambient (*Iridaea tuberculosa*). Another “unidentified” UV-absorbing compound was also found. The identification of MAAs by HPLC using secondary standards agreed well with HPLC-ESI-MS, mainly in *Nothogenia fastigiata* (palythinol, porphyra-334, shinorine, and asterina-330). For *Corallina officinalis*, the techniques differed by the identification of only one MAA, i.e., palythine (HPLC) and palythine-serine (HPLC-ESI-MS). However, for *I. tuberculosa*, HPLC-ESI-MS turned out to be a good tool because three more MAAs were recorded (palythine-serine, mycosporine glutamic acid, and mycosporine-glycine), including an unidentified potential MAA, as noted above. Shinorine, porphyra-334, palythine, and asterina-330 are the most common MAAs described in seaweeds [8], while mycosporine-glycine, mycosporine-glutamic acid, and palythine-serine are less frequent. In fact, palythine-serine had not been previously reported in seaweeds. 

Palythine-serine was previously described in corals [22,56,57,58,59], cyanobacteria [60,61,62], and in dinoflagellates [6]. Although the wavelength of the absorption maximum of palythine-serine and palythine are similar (λ_max_ = 320 nm), palythine-serine would be a secondary MAA synthesized from shinorine after decarboxylation and demethylation of the glycine subunit, as suggested in the literature [57,58], whereas palythine would be synthetized from a more primary MAA: mycosporine-glycine [6]. In the cyanobacterium *A. variabilis* PCC 7937, the synthesis of palythine-serine could be regulated by sulfur deficiency [60,61].

To the best of our knowledge, mycosporine-glutamic acid was reported a few times in the literature, mainly in fungal organisms, e.g., *Glomerella cingulate* [63] and *Heluella leucomelaneue* [64] but more recently in the red seaweed *Bostrychia scorpioides* from the coast of France [31]. Mycosporine-glutamic acid is a quantitatively less important MAA in the species studied, and its contribution to UV protection and as an antioxidant agent is still unknown. According to Wada et al. [65], the reaction of mycosporine-glutamic acid with singlet oxygen can be expected, though not much experimental detail has been reported [63].

According to its characteristics (λ_max_: 330 nm; *m*/*z*: 261.14189), the unidentified UV-absorbing compound could correspond to an aplysiapalythine C. However, further studies should be conducted for better identification. Aplysiapalythine A, B, and C were reported for the first time in the sea hare *Aplysia californica* [66], and the authors suggested that these marine animals acquire MAAs from their algal diet. More recently, Orfanoudaki et al. [19] reported aplysiapalythine A and B, but not aplysiapalythine C, in several seaweed species from New Zealand and Australia. The same authors explain that aplysiapalythine A and B were identified from MAA-enriched extracts and in trace concentrations. 

### 3.3. The Subantarctic Red Seaweeds as Source for Cosmetic Application

Currently, a few red seaweeds, such as *Porphyra* spp., *Bostrychia* spp., and *Bangia* spp. with high MAA concentration, supply the biotechnological industry with compounds to produce sunscreens [67,68,69]. Still, such compounds have yet to be fully exploited because of the inherent difficulty of ensuring sufficient algal biomass for MAAs extraction [70]. In the case of *Porphyra* spp., the alternation between macroscopic and microscopic phases makes it difficult to get sufficient algal biomass throughout the year, particularly in cold waters. In this sense, the diversification of sources for MAAs extraction among various seaweed species (e.g., *N. fastigiata* and *I. tuberculosa*) could result in a plentiful supply of algal biomass for extraction of these compounds, mainly in seasons when *Porphyra* spp. are in microscopic stages. In the case of *C. officinalis*, its low MAA concentration does not allow it to be used as source of MAAs; however, further investigation is warranted because many extracts of this species are now being used in the cosmetic industry based on its diverse properties, such as anti-aging, anti-inflammation, smoothing agent, and UV filter [71,72]. 

Even though the three species studied in the present work have a low concentration of MAAs when compared to *Porphyra* spp., they have the ability to modify not only their concentration but also their composition, depending on the season. Thus, modifying the factors in controlled culture systems could make it possible to change the MAA composition and content. Our research group reported the increase of MAA content in seaweeds cultured in tanks under controlled conditions [39,45,46,52].

Such diversification not only implies finding species with high MAA concentration but also species with different MAA composition or species with novel MAAs. In this respect, although low in concentration, *I. tuberculosa* possesses two MAAs uncommon in seaweeds, namely mycosporine-glutamic acid and palythine-serine. The ecophysiological and biotechnological relevance of these MAAs is still uncertain and further research is necessary. A detailed screening of MAAs and their biological activity should be carried out in seaweeds of the extreme environments. Because these organisms inhabit extreme environmental conditions, they could be an excellent source for ecologically and pharmacologically relevant natural compounds.

## 4. Material and Methods

### 4.1. Determination of Abiotic Settings in Bahía Mansa (Magellan Strait)

Surface water temperature was obtained from NASA satellite MODIS AQUA 4 km pixel size (average ± SD. N = 5–12) [73]. (These values were ground-validated with data collected from the HOBO Data logger (Onset Computer Corporation, Bourne, MA, USA) placed at the collection site during the year. Data on UV (W m^−2^) and PAR (μmol photon m^−2^ s^−1^) were obtained from the TUV model developed by Madronich et al. [74], considering measurements made around noon (17:00 h) during all seasons of 2018. 

### 4.2. Biological Material

Thalli of *Nothogenia fastigiata*, *Iridaea tuberculosa* and *Corallina officinalis* were collected from mid to lower intertidal of Bahía Mansa (53°36’ S; 70°55´ W: in Magellan Strait, Subantarctic area) throughout 2018. After collection, thalli were transported to the Laboratorio de Ecofisiología y Biotecnología de Alga (LEBA) located at the University of Magallanes, Punta Arenas, Chile. Each species, separated and identified, was dried in plastic bags containing silica gel and stored in a cool, dark, and dry place until analysis.

### 4.3. Extraction and Isolation; Spectrophotometric Analysis 

The UV-absorbing compounds were extracted in methanol (20%) at 45 °C during 2 h. After centrifugation (100 rpm during 10 min), the methanolic extracts were analyzed in a spectrophotometer (290–400 nm, Spectroquant^®^ Pharo 300). 

### 4.4. MAAs Extraction and Quantification by HPLC

MAAs were extracted in 1 mL of 20% aqueous methanol (*v*/*v*) for 2 h at 45 °C. After extraction, 600 mL of the supernatant were evaporated under vacuum to dry. Dried extracts were resuspended in 700 µL of 100% chromatographic methanol, followed by filtration through a 0.22 μm membrane filter. MAAs were detected by HPLC, using a flow isocratic gradient (1 mL/min) containing 1.5% aqueous methanol (*v*/*v*) and 0.15% acetic acid (*v*/*v*). Twenty μL of the sample filtrated was injected into a Sphereclone C8 column (5 μm particle size and 250 × 4.6 mm diameter), and a pre-column (Phenomenex, Aschaffenburg, Germany) was coupled to a Waters (Barcelona, Spain) HPLC system, according to [75] and modified by [20]. MAAs were detected with a Waters Photodiode Array Detector (996; Barcelona, Spain) at a wavelength of 330 nm. Absorption spectra were recorded between 290 and 400 nm. Identification of MAAs was performed by comparison of the absorption spectra and retention times with various well-characterized standards (*Mastocarpus stellatus*, *Bostrychia scorpioides*, and *Porphyra yezoensis*). The quantification followed the method described by [20], and the results were expressed as mg g^−1^ DW. 

### 4.5. MAAs Identification by High-Resolution Mass Spectra (HPLC-ESI-MS)

The identification of MAAs was carried out using HPLC (Ultimate 3000) equipped with thermostated autosampler (5 °C) and column (30 °C) (Thermo Scientific). The HPLC was coupled to a mass spectrometer (Orbitrap Q-Exactive) with atmospheric pressure ionization system and heated electrospray H-ESI (Thermo Scientific). The column used was C8 4 µM, 250 X 4.6 mm (Luna Phenomenex, Aschaffenburg, Germany). The fluxes of the mobile phases (A: H_2_O + 5 mM of NH_4_ at pH 4.5; B: acetonitrile) and the gradient used are shown in Table 2. The parameters of the mass spectrometer were adjusted as follows: resolution = 70.000 FWHM; Sheath gas = 40; Spray Voltage = 4 KV; Capillary Temperature = 320 °C; and S-Lens level = 55. Mass spectra were acquired at a 100–1200 m/z interval in positive and negative mode.

### 4.6. Statistical Analysis

The effects of seasonality on total MAA content and composition in *N. fastigiata*, *I. tuberculosa*, and *C. officinalis* were assessed using ANOVA [76]. For that purpose, one factor was considered: seasonality (fixed with 4 levels). Student Newman–Keuls tests (SNK) were performed after significant ANOVAs [76]. Homogeneity and homoscedasticity of variance were tested using Cochran tests and by visual inspection of the residuals. Analyses were performed using SPSS v.21 (IBM). The Pearson correlation coefficients were calculated and tested among all variables. Analyses were performed using SigmaPlot 12.0 (Systat software, Inc., San Jose California USA).

## 5. Conclusions

*Nothogenia fastigiata*, *Iridaea tuberculosa*, and *Corallina officinalis* exhibited a different seasonal dynamic in MAAs accumulation. This dynamic is not related to the seasonality of solar radiation itself but rather to variations of different factors at local levels. Seven MAAs were identified, and one unidentified UV-absorbing compound was recorded. Two MAAs were recorded for the first time in seaweeds from Subantarctic areas: mycosporine-glutamic acid and palythine-serine. These two MAAs were identified in *I. tuberculosa*, which also exhibited the greatest total MAA concentration among the studied species. Finally, we found that the seaweeds from high latitude have the ability to accumulate and vary MAA content and composition. This can have important implications for the use of these seaweeds as alternative sources of MAAs for the biotechnological and pharmacology industries.

## Figures and Tables

**Table 1:** The chemical formula of MAAs identified by high-resolution mass spectra/HPLC-ESI-MS. RT: Retention Time (min).

	Formula	λmax	Accuracy (ppm)	*m*/*z*	RT	Species
Porphyra-334	C_14_H_22_N_2_O_8_	334	1.2	347.14142	5.85	*N. fastigiata*
	C_14_H_22_N_2_O_8_		0.8	347.13842	5.87	*I. tuberculosa*
	C_14_H_22_N_2_O_8_		0.5	347.14142	5.87	*C. officinalis*
Asterina-330	C_12_H_20_N_2_O_6_		1	289.13652	5.73	*N. fastigiata*
	C_12_H_20_N_2_O_6_	330	0.2	289.13652	6	*I. tuberculosa*
	C_12_H_20_N_2_O_6_		0.1	289.13652	6.01	*C. officinalis*
Shinorine	C_13_H_20_N_2_O_8_		0.6	333.12591	5.89	*N. fastigiata*
	C_13_H_20_N_2_O_8_	334	0.5	333.12591	5.89	*I. tuberculosa*
	C_13_H_20_N_2_O_8_		0.6	333.12591	5.89	*C. officinalis*
Palythinol	C_13_H_22_N_2_O_6_	332	0.8	303.15203	6.14	*N. fastigiata*
	C_13_H_22_N_2_O_6_		0.01	303.15203	6.16	*I. tuberculosa*
Palythine-Serine	C_11_H_18_N_2_O_6_	320	0.3	275.1201	5.83	*I. tuberculosa*
	C_11_H_18_N_2_O_6_		0.1	275.1201	5.83	*C. officinalis*
Mycosporine-Glycine	C_10_H_15_NO_6_	310	0.4	246.09475	2.91	*I. tuberculosa*
Mycosporine-glutamic acid	C_13_H_19_N0_8_	311	0.1	318.11516	5.56	*I. tuberculosa*
Unidentified UVAC	C_11_H_21_N_2_0_5_	330	0.1	261.14189	5.79	*I. tuberculosa*

**Table 2:** Fluxes of the mobile phases (A = 5 mM of NH_4_ at pH 4.5; B = acetonitrile) and the gradient used to identify MAAs by high-resolution mass spectra (HPLC-ESI-MS).

Time (min)	Flux (mL/min)	A%	B%
0	0.2	10	90
0.3	0.2	10	90
1	0.2	40	60
3	0.2	40	60
3.5	0.2	95	5
7	0.2	95	5
7.5	0.2	10	90
11.5	0.2	10	90

## Supplementary Materials

The following are available online at https://www.mdpi.com/1660-3397/18/2/75/s1, Table S1: ANOVA results testing the season effect on MAAs total accumulation, Table S2.A: Person correlation between Solar radiation; in UVB, UVA and PAR and total MAAs in *Nothogenia fastigiata*, Table S2.B: Person correlation between Solar radiation; in UVB, UVA and PAR and total MAAs in *Iridaea tuberculosa,* Table S2.C: Person correlation between Solar radiation; in UVB, UVA and PAR and total MAAs in *Corallina officinalis*, Table S2: ANOVA results testing season effect on MAA composition, Figure S1: Absorption maxima and molecular mass of Asterine-330 in in *Nothogenia fastigiata*, Figure S2: Absorption maxima and molecular mass of Shinorine in *Nothogenia fastigiata,* Figure S3: Absorption maxima and molecular mass of Porphyra-334 in *Nothogenia fastigiata,* Figure S4: Absorption maxima and molecular mass of Palythinol in *Nothogenia fastigiata*, Figure S5: Absorption maxima and molecular mass of Mycosporine-Gly in *Iridaea tuberculosa,* Figure S6: Absorption maxima and molecular mass of Mycosporine-Glutamic acid in *Iridaea tuberculosa*, Figure S7: Absorption maxima and molecular mass of a unidentified UV-Absorbing compound, Figure S8: Absorption maxima and molecular mass of Palythine-Serine in *Iridaea tuberculosa,* Figure S9: Absorption maxima and molecular mass of Porphyra-334 in *Iridaea tuberculosa,* Figure S10: Absorption maxima and molecular mass of Palythinol in *Iridaea tuberculosa,* Figure S11: Absorption maxima and molecular mass of Asterine-330 in *Iridaea tuberculosa,* Figure S12: Absorption maxima and molecular mass of Palythine-Serine in *Corallina officinalis,* Figure S13: Absorption maxima and molecular mass of Asterine-330 in *Corallina officinalis,* Figure S14: Absorption maxima and molecular mass of Porphyra-334 in *Corallina officinalis*, Figure S15: Absorption maxima and molecular mass of Shinorine in *Corallina officinalis*.
