# Supplementary material for: Seasonal Variation of Mycosporine-Like Amino Acids in Three Subantarctic Red Seaweeds
Source: Mar Drugs. 2020 Jan 24;18(2):75. doi: 10.3390/md18020075 (PMC7074333; doi:10.3390/md18020075)

SUPPLEMENTARY MATERIAL

Table S1, ANOVA results after seasonal experiment testing for the effect of season on the MAAs total accumulation in *Nothogenia fastigiata, Iridaea tuberculosa* and *C. officinalis,* $p<0.05$**

		Total MAAs			
		df	*MS*	*F*	*P*
N. fastigiata	*season*	3	**1.794823687**	**65.97504932**	**
	Res	8	0.027204583		
I. tuberculosa	*season*	8	**2264.59**	**3952.9**	**
	Res	18	0.57		
C. officinalis	*season*	8	**3092.48**	**15272.6**	**
	Res	18	0.2		

Res: Residual

Table S2.A Person correlation between Solar radiation; in UVB, UVA and PAR and total MAAs in *Nothogenia fastigiata* along to the seasonal time.

N. fastigiata	UVB	UVA	PAR
**Total MAAs**	0,814	0,810	0,809
	**0,00129**	**0,00139**	**0,00143**
**UVB**		1,000	1,000
		**4,014E-023**	**6,037E-022**
**UVA**			1,000
			**3,494E-028**

Table S2.B Person correlation between Solar radiation; in UVB, UVA and PAR and total MAAs in *Iridaea tuberculosa,* along to the seasonal time.

I. tuberculosa	UVB	UVA	PAR
**Total MAAs**	-0,104	-0,106	-0,107
	0,747	0,742	0,740
**UVB**		1,000	1,000
		**4,014E-023**	**6,037E-022**
**UVA**			1,000
			**3,494E-028**

Table S2.C Person correlation between Solar radiation; in UVB, UVA and PAR and total MAAs in *Corallina officinalis* along to the seasonal time.

C. officinalis	UVB	UVA	PAR
**Total MAAs**	0,790	0,792	0,793
	**0,00221**	**0,00214**	**0,00211**
**UVB**		1,000	1,000
		**4,014E-023**	**6,037E-022**
**UVA**			1,000
			**3,494E-028**

Table S2, ANOVA results after seasonal experiment testing for the effect of season on the MAAs content in *N. fastigiata, I. tuberculosa* and *C. officinalis* sp. *p*<0.05**

			MAAs contents			
			df	*MS*	*F*	*P*
N. fastigiata	*%Palithynol*	*Season*	3	433	2.6	0.12
		Res	8	165		
	%Porphyra 334	*Season*	3	423	2.7	0.11
		Res	8	154		
	%Shinorine	*Season*	3	1.5	3.5	0.07
		Res	8	0.4		
	%Asterine 330	*Season*	**3**	**18**	**132**	**
		Res	8	0.1		
	%Palythine	*Season*	**3**	**0.03**	**26**	**
		Res	8	0.001		
I. tuberculosa	*%Palithynol*	*Season*	3	433	2.6	0.12
		Res	8	165		
	%Porphyra 334	*Season*	3	*nd*	*nd*	*nd*
		Res	8			
	%Shinorine	*Season*	3	**520**	**34.4**	**
		Res	8	15		
	%Asterine 330	*Season*	3	**0.81**	**9.7**	**
		Res	8	0.08		
	%Palythine	*Season*	3	91	3.2	0.09
		Res	8	28.		
C. officinalis	*%Palithynol*	*Season*	3	**215**	**7.0**	**
		Res	8	30		
	%Porphyra 334	*Season*	3	46	3.3	0.08
		Res	8	13		
	%Shinorine	*Season*	3	285	2.5	0.13
		Res	8	114		
	%Asterine 330	*Season*	3	3.4	0.9	0.45
		Res	8	3.5		
	%Palythine	*Season*	3	22	0.9	0.45
		Res	8	22		

Res: Residual; nd: no data

Figure S1.- Mycosporine like amino acid in *Nothogenia fastigiata,* Asterine-330

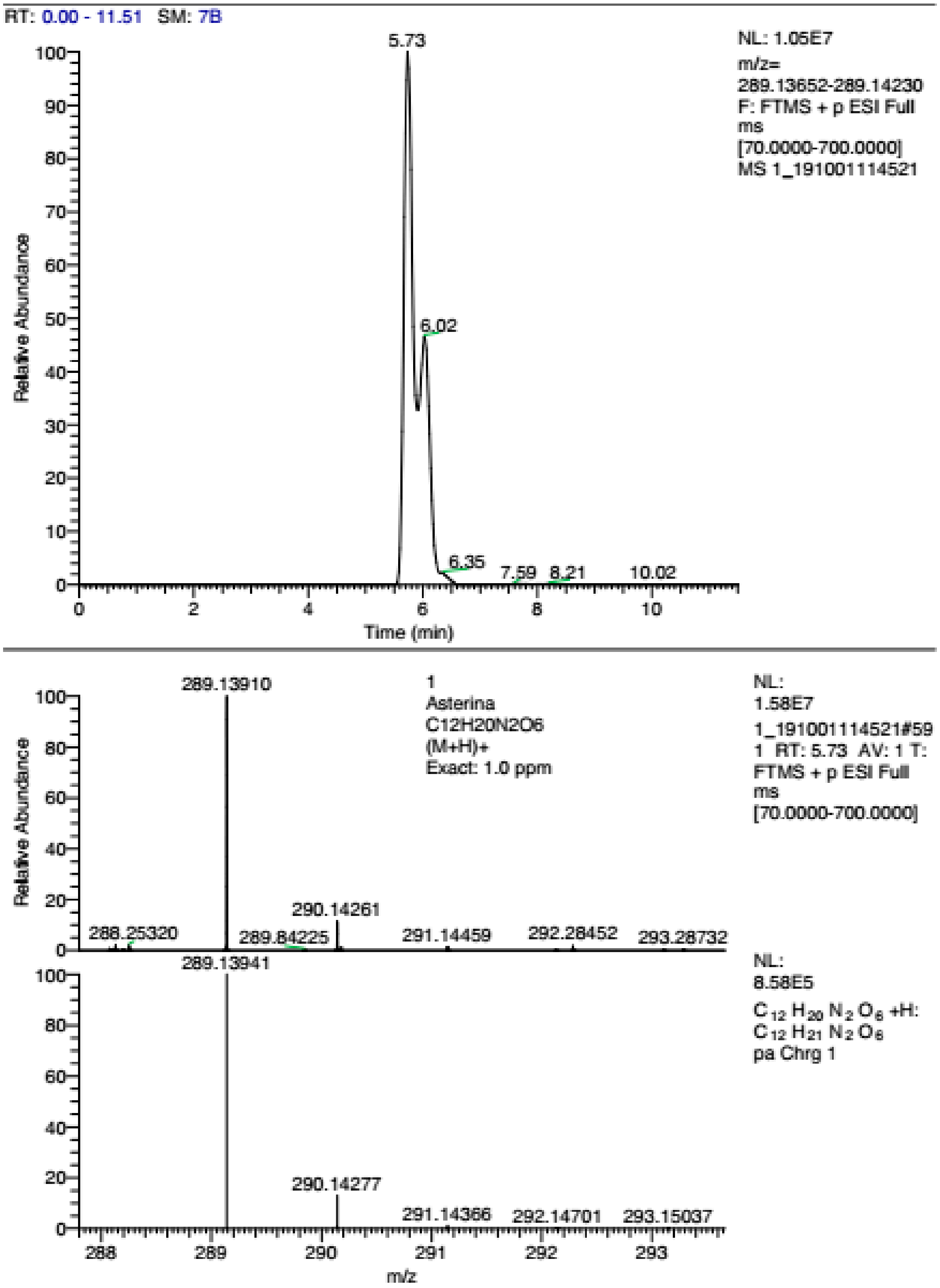

Figure S2.- Mycosporine like amino acid in *Nothogenia fastigiata*, Shinorine

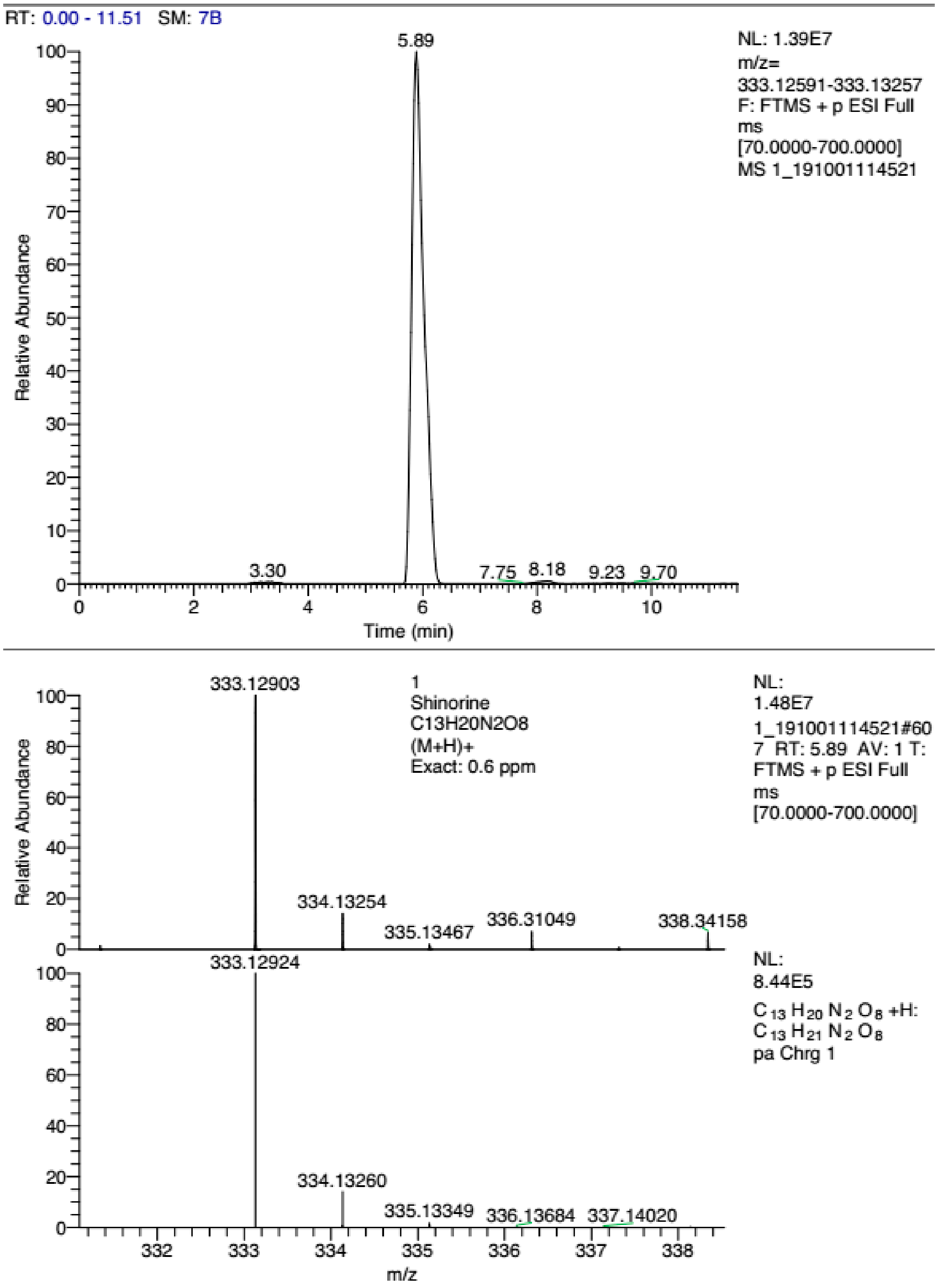

Figure S3.- Mycosporine like amino acid in *Nothogenia fastigiata*, Porphyra-334

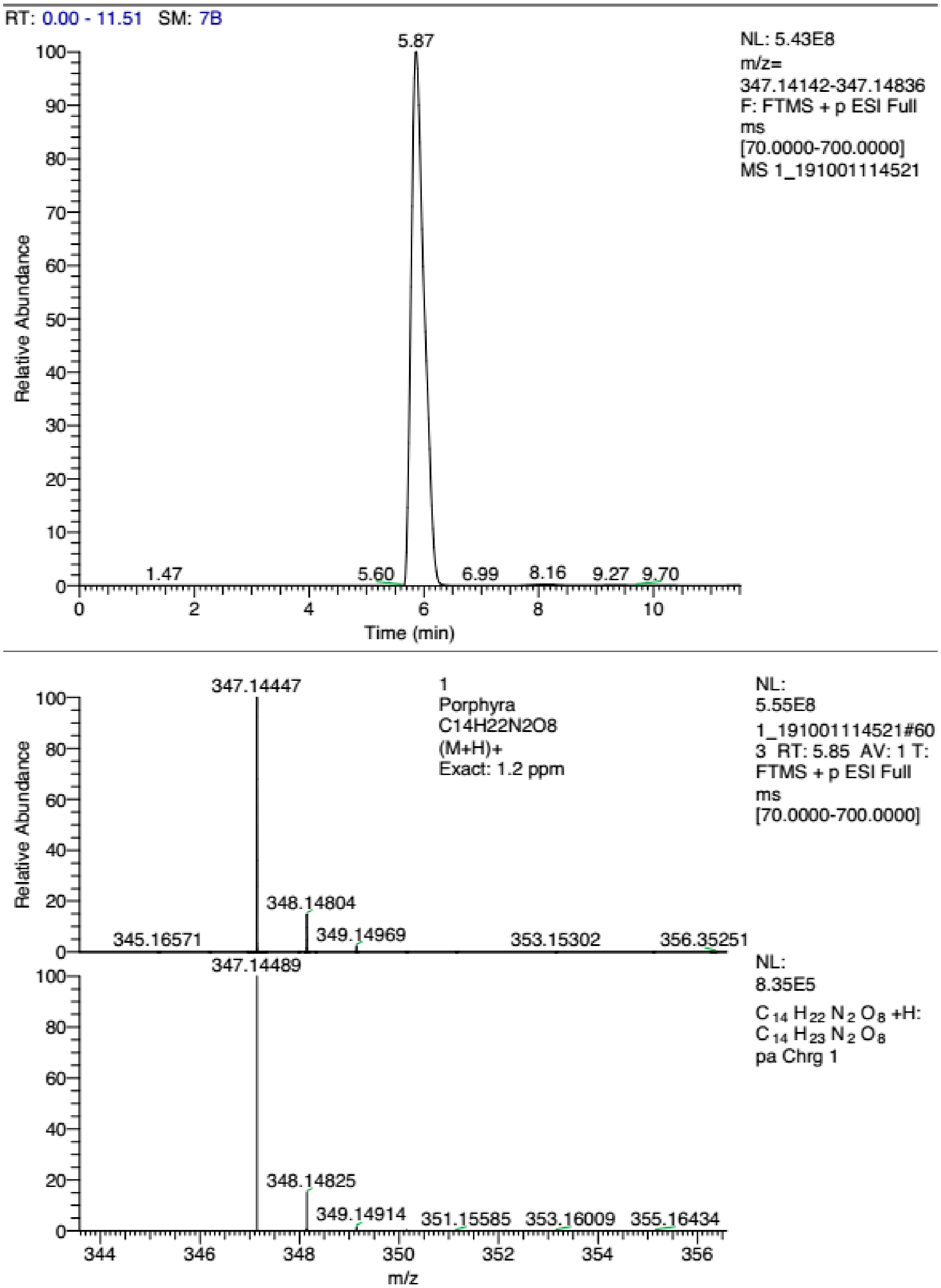

Figure S4.- Mycosporine like amino acid in *Nothogenia fastigiata*, Palythinol

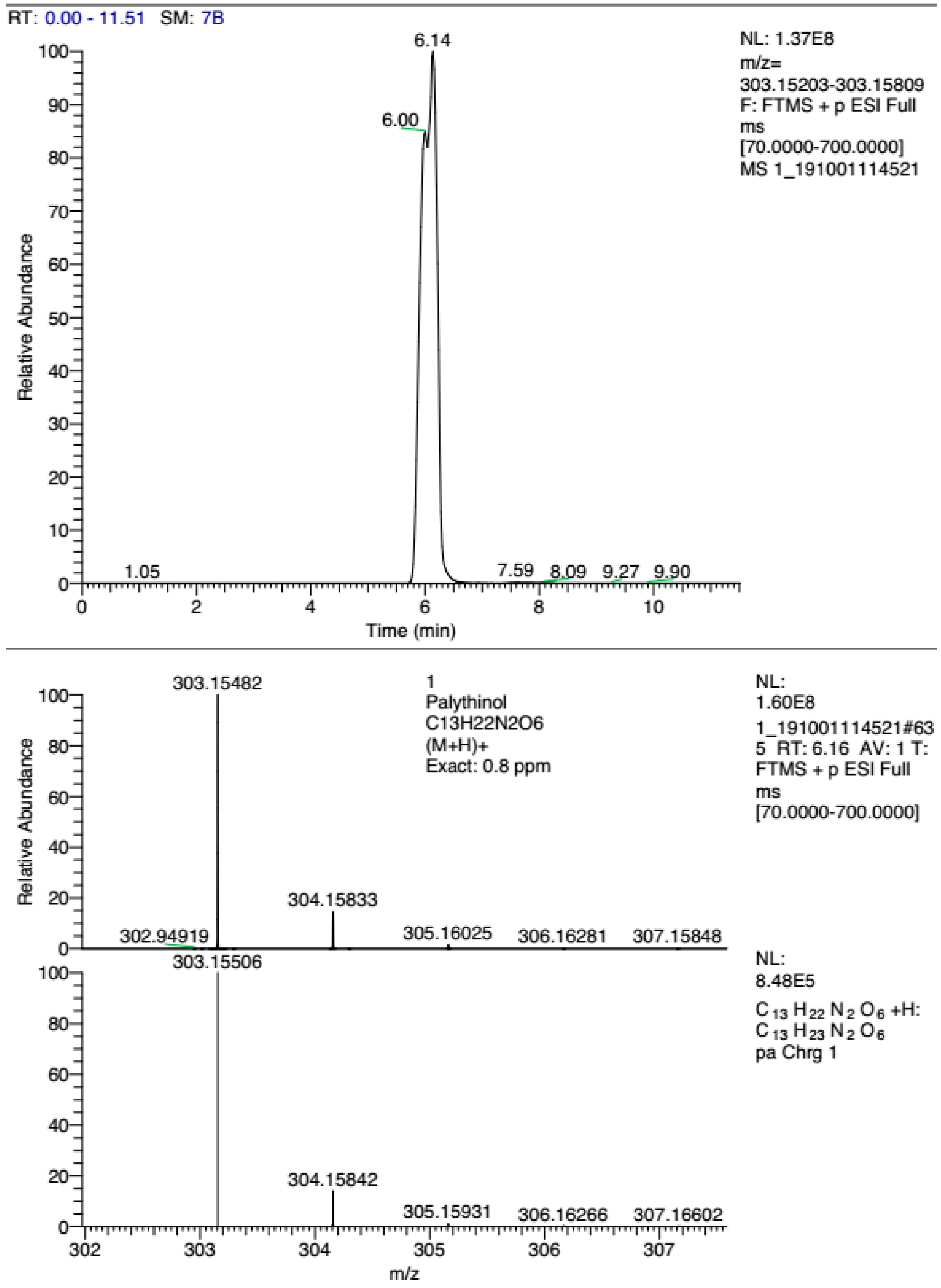

Figure S5.- Mycosporine like amino acid in *Iridaea tuberculosa*, Mycosporine-Gly

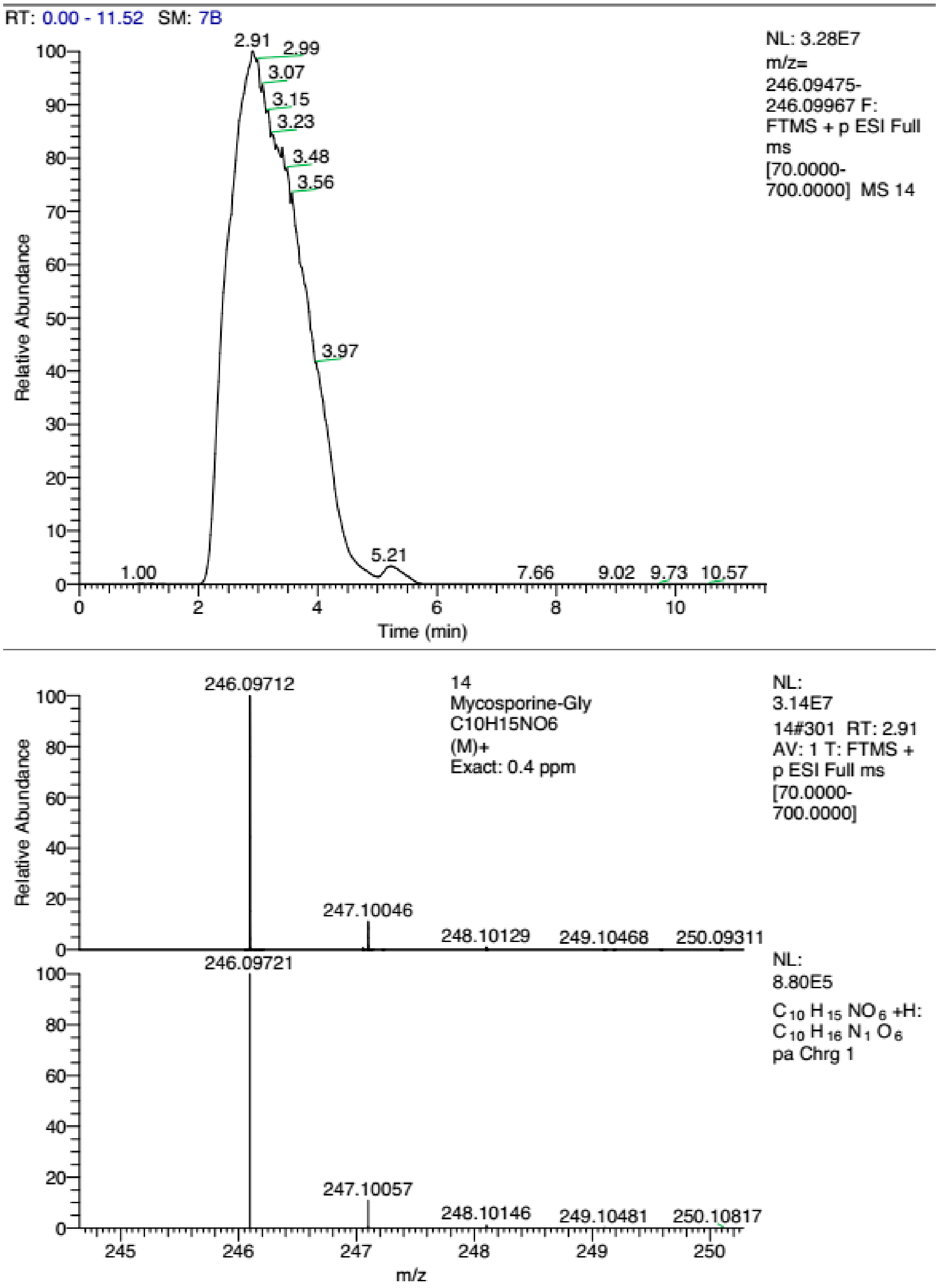

Figure S6.- Mycosporine like amino acid in *Iridaea tuberculosa,* Mycosporine-Glutamic acid

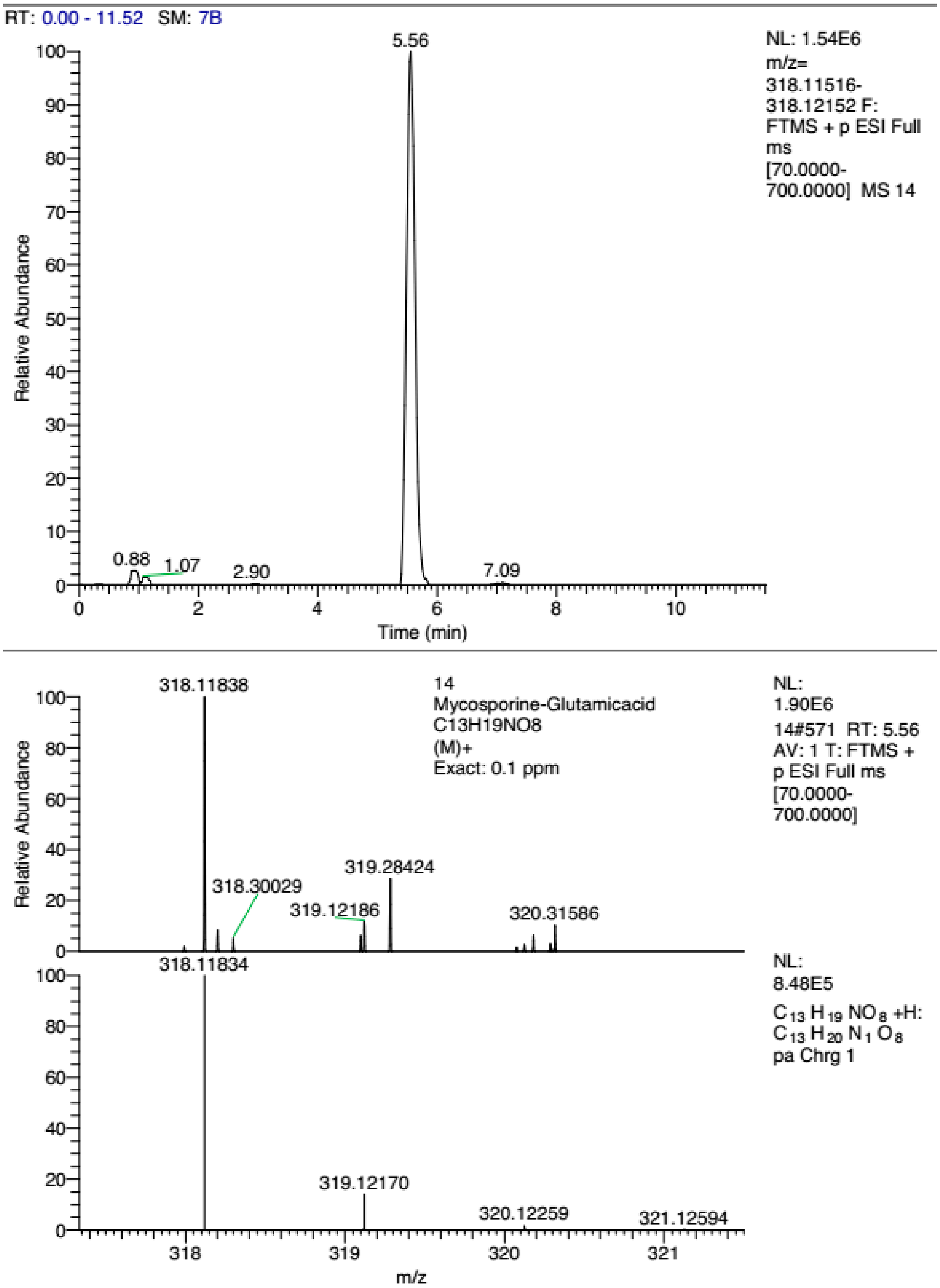

Figure S7.- Mycosporine like amino acid in *Iridaea tuberculosa*, UV-Absorbing compound non-identified

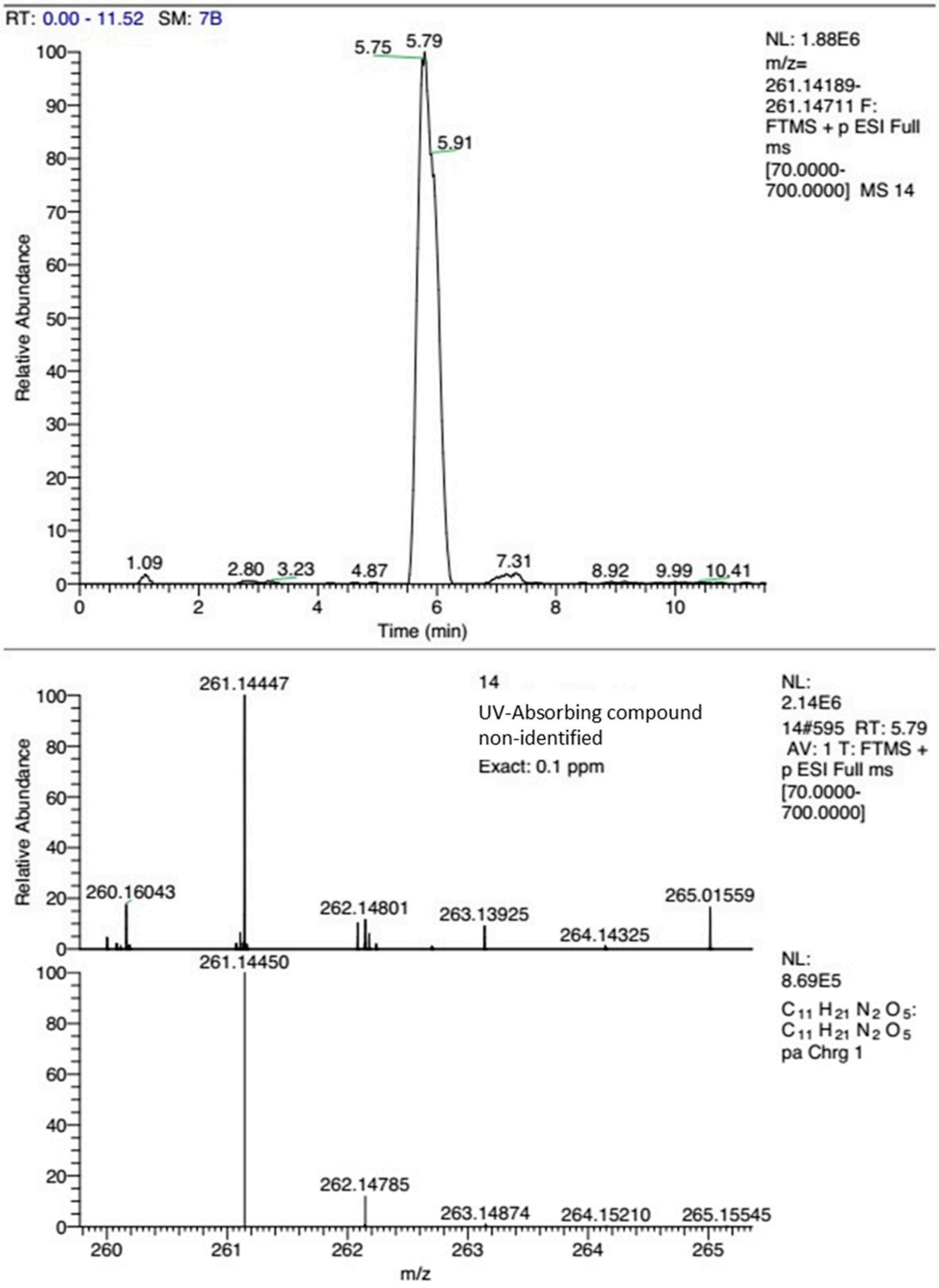

Figure S8.- Mycosporine like amino acid in *Iridaea tuberculosa,* Palythine-Serine

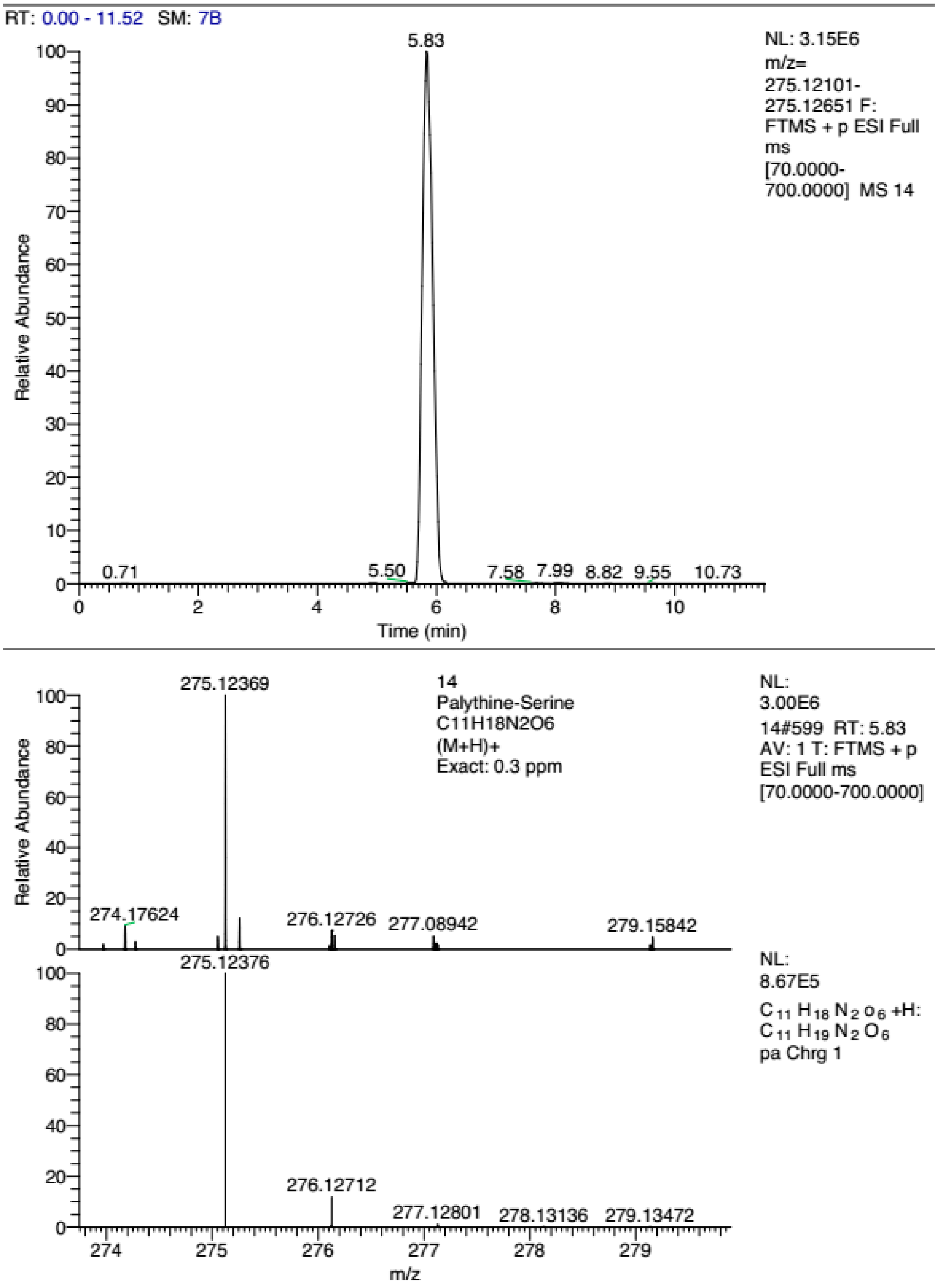

Figure S9.- Mycosporine like amino acid in *Iridaea tuberculosa,* Porphyra-334

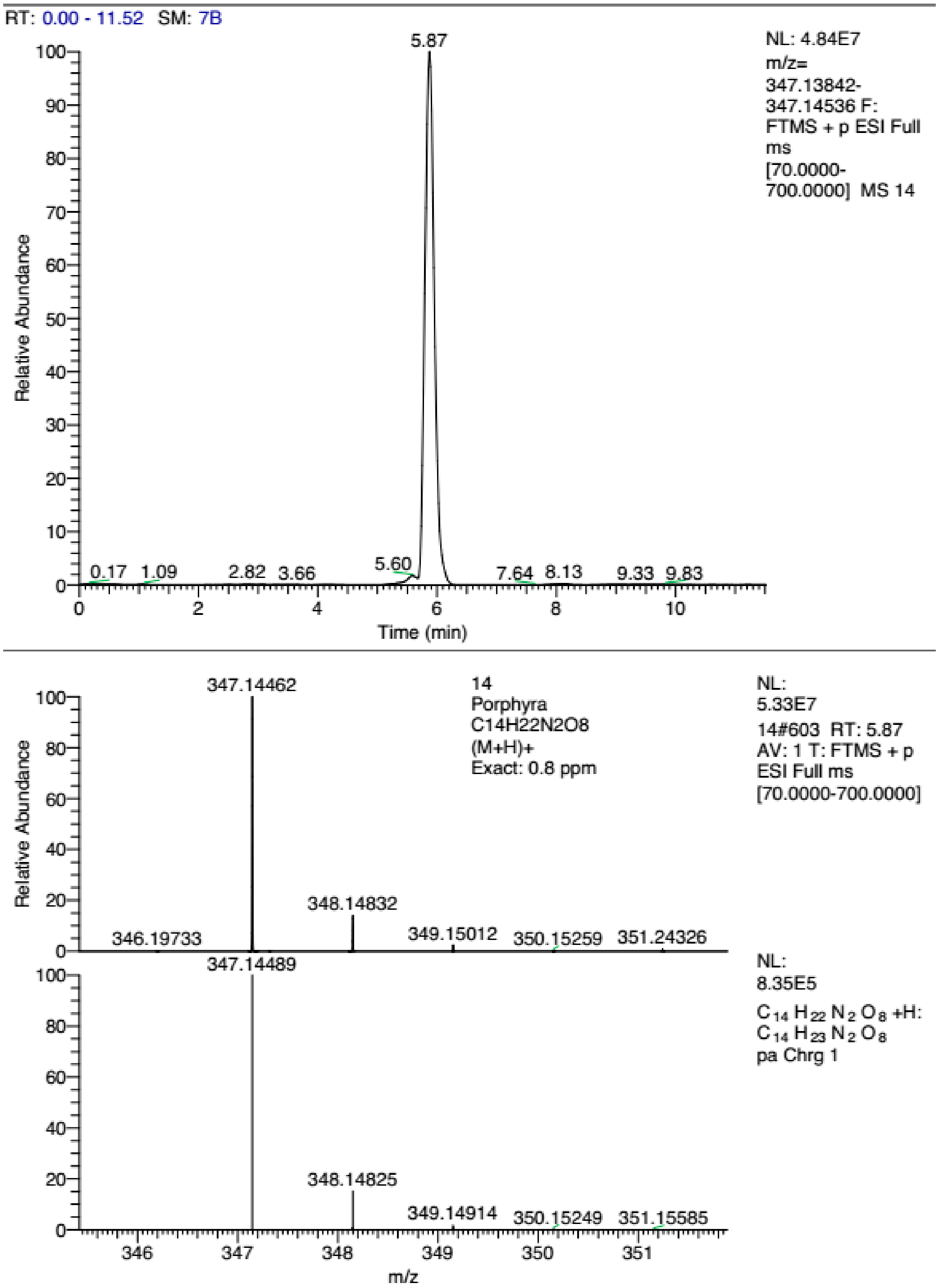

Figure S10.- Mycosporine like amino acid in *Iridaea tuberculosa*, Palythinol

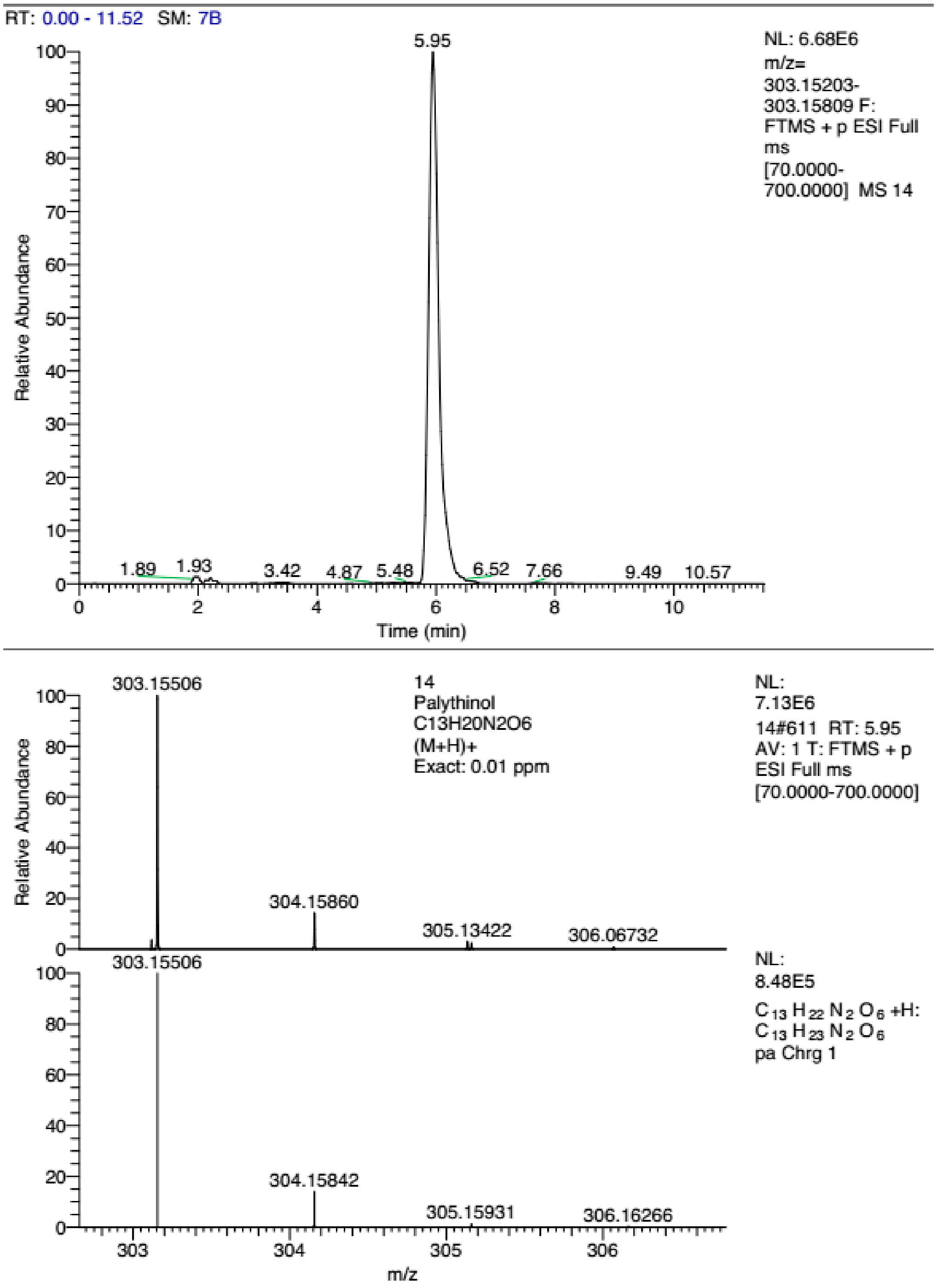

Figure S11.- Mycosporine like amino acid in *Iridaea tuberculosa*, Asterine-330

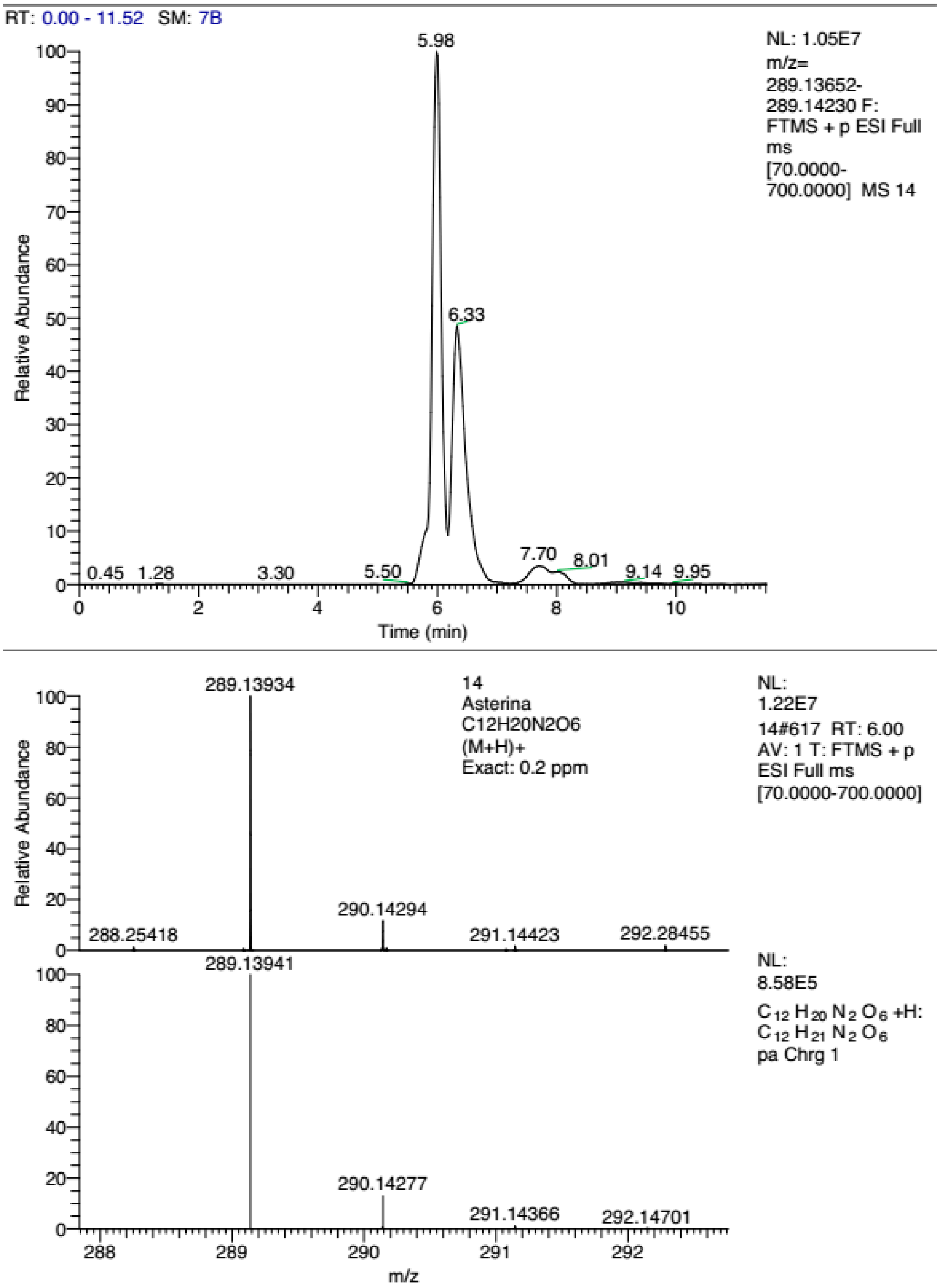

Figure S12.- Mycosporine like amino acid in *Corallina officinalis*, Palythine-Serine

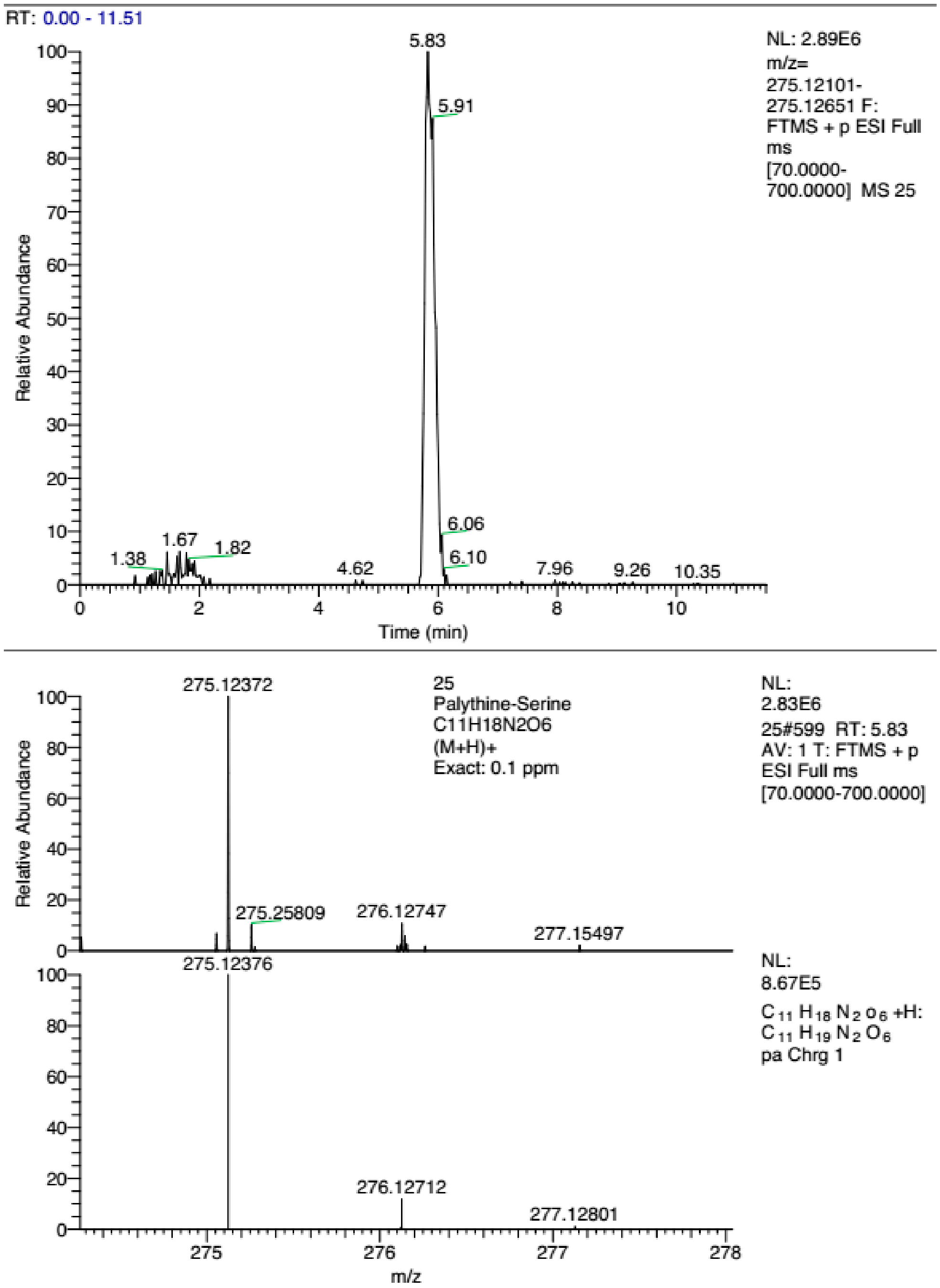

Figure S13.- Mycosporine like amino acid in *Corallina officinalis*, Asterine-330

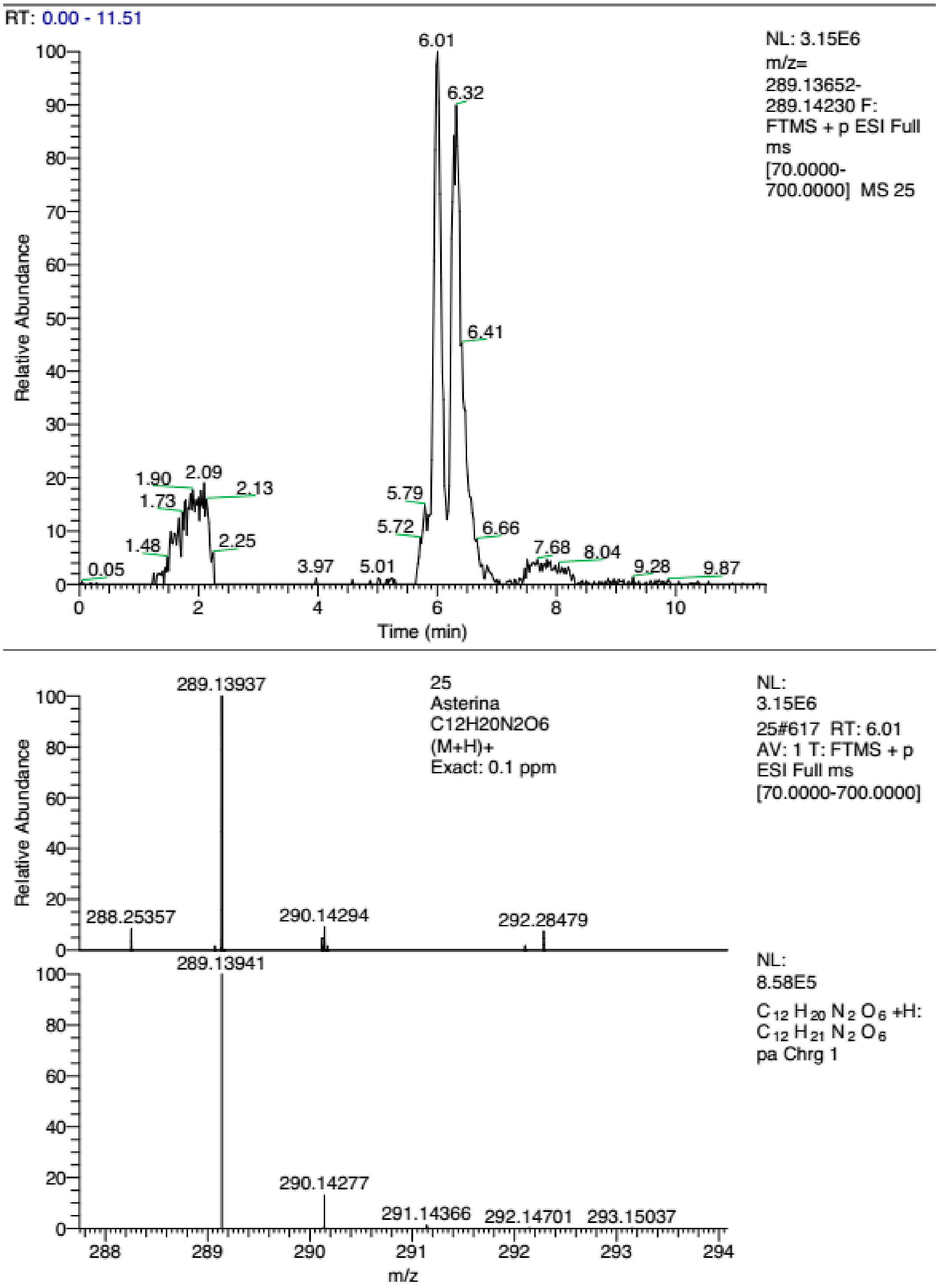

Figure S14.- Mycosporine like amino acid in *Corallina officinalis*, Porphyra-334

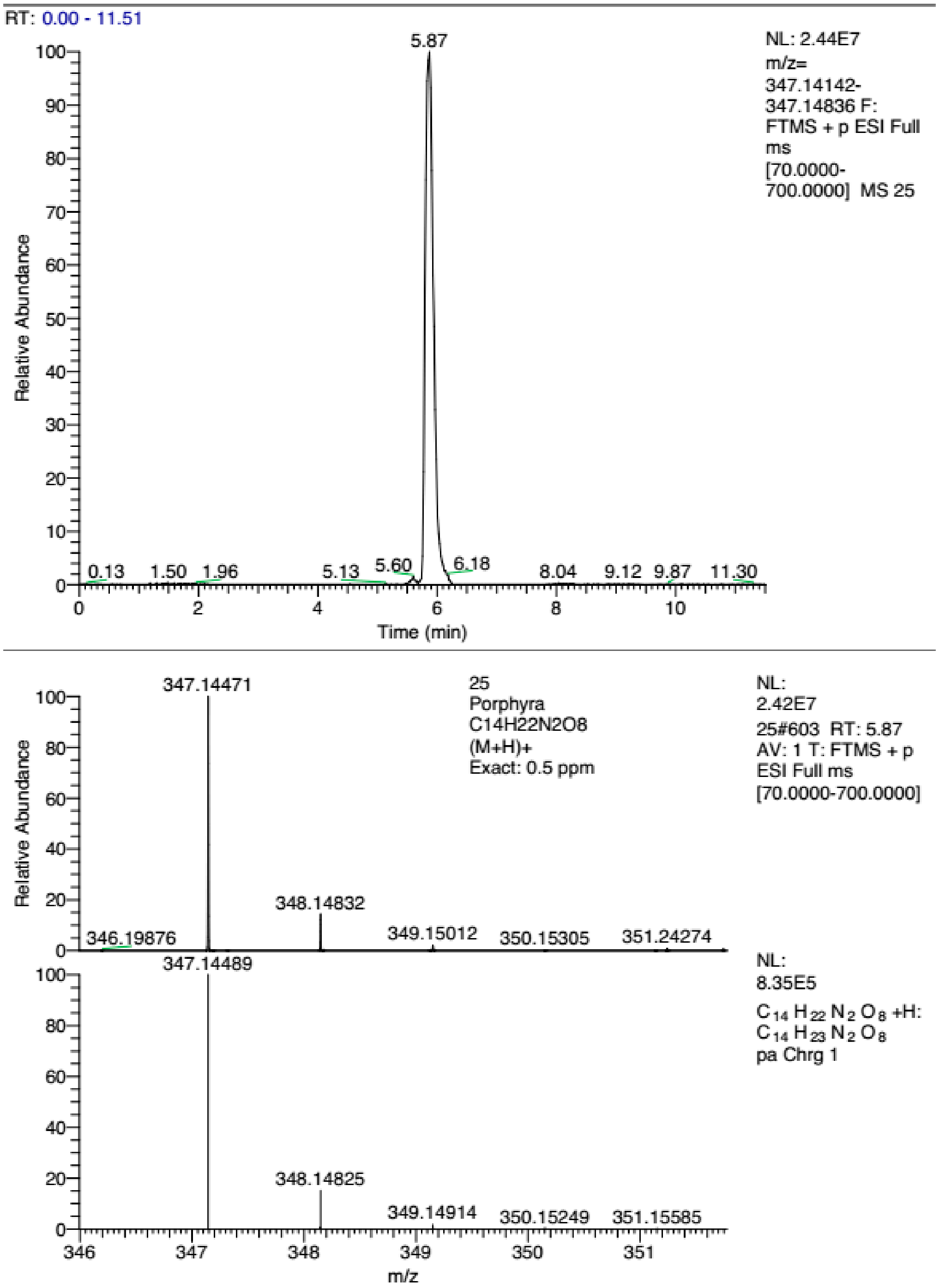

Figure S15.- Mycosporine like amino acid in *Corallina officinalis*, Shinorine

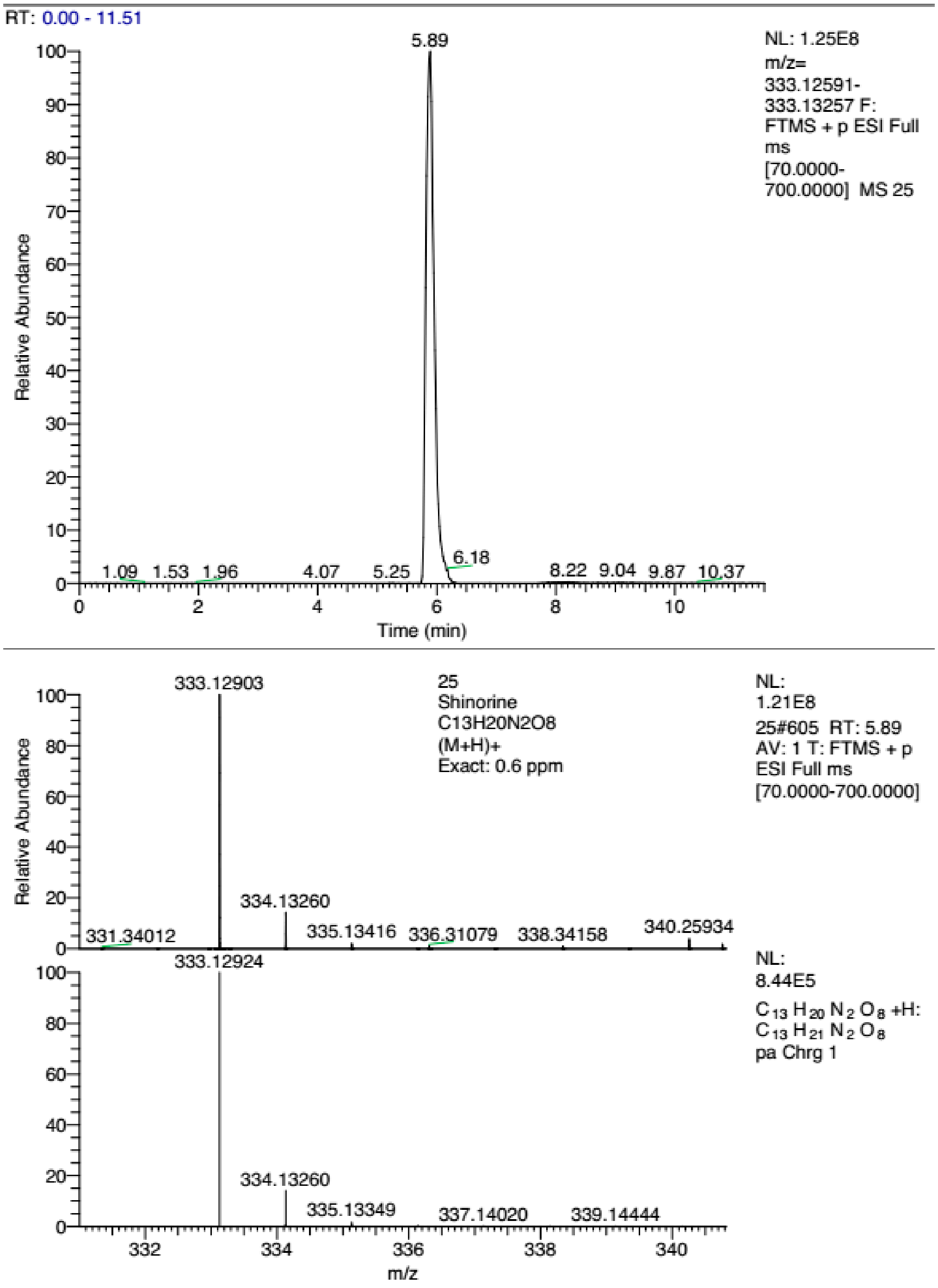

Supplement: Supplementary file 1 [file marinedrugs-18-00075-s001.pdf]
